# Modeling of lifetime scenarios with non-monotonic failure rates

**DOI:** 10.1371/journal.pone.0314237

**Published:** 2025-01-22

**Authors:** Amani Abdullah Alahmadi, Olayan Albalawi, Rana H. Khashab, Arne Johannssen, Suleman Nasiru, Sanaa Mohammed Almarzouki, Mohammed Elgarhy

**Affiliations:** 1 College of Science and Humanities, Shaqra University, Shaqra, Saudi Arabia; 2 Department of Statistics, Faculty of Science, University of Tabuk, Tabuk, Saudi Arabia; 3 Mathematics Department, Faculty of Sciences, Umm Al-Qura University, Makkah, Saudi Arabia; 4 Faculty of Business Administration, University of Hamburg, Hamburg, Germany; 5 Department of Statistics and Actuarial Science, School of Mathematical Sciences, C. K. Tedam University of Technology and Applied Sciences, Navrongo, Ghana; 6 Statistics Department, Faculty of Science, King Abdul Aziz University, Jeddah, Kingdom of Saudi Arabia; 7 Mathematics and Computer Science Department, Faculty of Science, Beni-Suef University, Beni-Suef, Egypt; 8 Department of Basic Sciences, Higher Institute of Administrative Sciences, Belbeis, AlSharkia, Egypt; Abdul Wali Khan University Mardan, PAKISTAN

## Abstract

The Weibull distribution is an important continuous distribution that is cardinal in reliability analysis and lifetime modeling. On the other hand, it has several limitations for practical applications, such as modeling lifetime scenarios with non-monotonic failure rates. However, accurate modeling of non-monotonic failure rates is essential for achieving more accurate predictions, better risk management, and informed decision-making in various domains where reliability and longevity are critical factors. For this reason, we introduce a new three parameter lifetime distribution—the Modified Kies Weibull distribution (MKWD)—that is able to model lifetime scenarios with non-monotonic failure rates. We analyze the statistical features of the MKWD, such as the quantile function, median, moments, mean, variance, skewness, kurtosis, coefficient of variation, index of dispersion, moment generating function, incomplete moments, conditional moments, Bonferroni, Lorenz, and Zenga curves, and order statistics. Various measures of uncertainty for the MKWD such as Rényi entropy, exponential entropy, Havrda and Charvat entropy, Arimoto entropy, Tsallis entropy, extropy, weighted extropy and residual extropy are computed. We discuss eight different parameter estimation methods and conduct a Monte Carlo simulation study to evaluate the performance of these different estimators. The simulation results show that the maximum likelihood method leads to the best results. The effectiveness of the newly suggested model is demonstrated through the examination of two different sets of real data. Regression analysis utilizing survival times data demonstrates that the MKWD model offers a superior match compared to other current distributions and regression models.

## 1 Introduction

Probability distributions form the foundation of parametric statistical analysis, and therefore researchers are constantly developing new distributions and/or modifying existing ones. For instance, the Weibull distribution (WD) [1] is one of the established distributions that has gained considerable attention in reliability analysis and lifetime modeling. The WD is a highly adaptable statistical tool extensively utilized in many sectors like engineering, health, finance, and environmental sciences. It is valued for its effectiveness in accurately representing dependability and failure statistics. Engineering relies on this tool to approximate the duration of product functionality and assess the likelihood of failures. Similarly, medicine uses it in survival analysis to anticipate patient outcomes. Finance uses it to evaluate financial risks, while environmental sciences employ it to simulate data such as rainfall and temperature extremes. The versatility of the WD makes it indispensable for data analysis and forecasting, with several research papers confirming its usefulness in various fields [2, 3]. The cumulative distribution function (cdf) and probability density function (pdf) of the WD are given by

$$\begin{array}{c} G(y;\beta ,\vartheta )=1-e^{-\beta y^{\vartheta }} \end{array}$$ (1)

and

$$\begin{array}{c} g(y;\beta ,\vartheta )=\beta \vartheta y^{\vartheta -1}e^{-\beta y^{\vartheta }}, \end{array}$$ (2)

*y* > 0, where *β* > 0 and *ϑ* > 0 are two scale and shape parameters, respectively. The WD is an extreme value distribution and frequently used in modeling extreme events. The practicality of the WD in reliability/survival analysis and in modeling of lifetime data is high due to its property in handling lifetime scenarios with decreasing, constant or increasing failure rates. However, it fails to offer a good fit to lifetime data that exhibits non-monotonic failure rates such as bathtub (for example, human life cycle) or upside-down bathtub (for example, machine life cycle). Due to the drawbacks of the WD, a number of variants of the distribution has been proposed in the literature with the goal of enhancing its modeling capabilities as well as making it suitable for specific modeling lifetime phenomena. There are some recent discussed variants, such as the exponentiated WD [4], transmuted additive WD [5], Kumaraswamy transmuted exponentiated modified WD [6], Topp-Leone Modified WD [7], Burr X exponentiated WD [8], Kavya-Manoharan exponentiated WD [9], Marshall-Olkin power-generalized WD [10], truncated Cauchy power Weibull-G [11], alpha power transformed Weibull-G [12], Weighted WD [13], exponentiated power generalized Weibull power series family [14], exponentiated truncated inverse Weibull-G [15], odd inverse power generalized WD [16], extended inverse WD [17], Weibull WD (WWD) [18], and exponentiated WWD [19].

However, it is still a challenge to develop a distribution that is well suited for modeling lifetime scenarios characterized by non-monotonic failure rates reflecting real-world situations where the failure rate of a system or component may vary over time. To motivate the need for this study, we briefly describe the reasons why it is necessary to have a distribution at hand that can model lifetime scenarios with non-monotonic failure rates in an appropriate way:

- As non-monotonic failure rates occur when the probability of failure changes over the lifetime of a system or component, a suitable distribution would allow for a more accurate representation of the actual failure behavior observed in many systems.
- Understanding the pattern of failure rates over time is crucial for assessing risks associated with a system. If failure rates are non-monotonic, there may be periods of increased risk followed by periods of decreased risk. Properly modeling these fluctuations helps in identifying and managing risks effectively.
- Reliability analysis involves predicting the likelihood of a system operating without failure over a certain period. By using a distribution that accounts for non-monotonic failure rates, engineers and analysts can more precisely estimate the reliability of a system and make informed decisions regarding maintenance, design improvements, or replacement strategies.
- In many industries, decisions regarding maintenance schedules, warranty policies, and product design depend heavily on accurate assessments of failure rates. Using a distribution that can handle non-monotonic failure rates ensures that these decisions are based on realistic expectations and reduce the likelihood of unexpected failures or unnecessary costs.
- Non-monotonic failure rates are common in various fields such as engineering, finance, healthcare, and beyond. Having a distribution that can accommodate these patterns makes it applicable across a wide range of industries and scenarios.

In this study, addressing the above aims, we develop a new three-parameter lifetime distribution, the so called Modified Kies Weibull distribution (MKWD) by utilizing the modified Kies (MK) family of distributions [20]. The cdf and pdf of the MK family of distributions are

$$\begin{array}{c} F\left(y;\varpi \right)=1-e^{-{\left[\frac{G(y;\varpi )}{1-G(y;\varpi )}\right]}^{\zeta }},\;\;\;\;\;\;\;y\in R,\;\;\zeta >0, \end{array}$$ (3)

and

$$\begin{array}{c} f\left(y;\varpi \right)=\frac{\zeta g(y;\varpi )G{\left(y;\varpi \right)}^{\zeta -1}}{{\left[1-G(y;\varpi )\right]}^{\zeta +1}}e^{-{\left[\frac{G(y;\varpi )}{1-G(y;\varpi )}\right]}^{\zeta }},\;\;\;\;\;\;y\in R,\;\;\zeta >0, \end{array}$$ (4)

respectively, where *g*(*y*; *ϖ*) and *G*(*y*; *ϖ*) are the parent pdf and cdf for the baseline distribution with set of parameters *ϖ*, and *ζ* being the shape parameter of the family. In addition, Al-Babtain et al. [20] utilized the binomial and exponential series to rewrite the pdf as a linear combination of the exponentiated family,

$$\begin{array}{c} f\left(y;\varpi \right)=g(y;\varpi )\sum_{i,j=0}^{\infty }\eta_{i,j}{\left[G(y;\varpi )\right]}^{\zeta (i+1)+j-1}, \end{array}$$ (5)

where $\eta_{i,j}=\frac{{\left(-1\right)}^{i}\zeta }{i!}(\begin{array}{c} \zeta (i+1)+j\\ j \end{array})$. To determine some measures of uncertainty, we determine the expansion of [*f*(*y*; *ϖ*)]^∇^ via

$$\begin{array}{c} {}[f(y;\varpi ){]}^{\nabla }=[g(y;\varpi ){]}^{\nabla }\sum_{i,j=0}^{\infty }{℘}_{i,j}G{\left(y;\varpi \right)}^{\nabla (\zeta -1)+\zeta i+j}, \end{array}$$ (6)

where ${℘}_{i,j}=\zeta^{\nabla }\frac{{\left(-1\right)}^{i}\nabla^{i}}{i!}(\begin{array}{c} \nabla (\zeta +1)+\zeta i+j-1\\ j \end{array})$.

As a result of the above discussion, this study addresses the following issues:

- We enhance the adaptability of the Weibull model by utilizing the MK family. To be more specific, declining, right-skewed and unimodal forms are denoted for the pdf but the hazard rate function (hrf) can be bathtub, declining, increasing and J-shaped for the MKWD.
- Some statistical properties of the MKWD, such as moments, mean, variance, skewness, kurtosis, coefficient of variation, index of dispersion, moment generating function, incomplete moments, conditional moments, Bonferroni (BON), Lorenz (LOR), and Zenga (ZEN) curves [21, 22], and order statistics (OS), are calculated.
- Various measures of uncertainty for the MKWD such as Rényi entropy (RE) [23], exponential entropy (EE) [24], Havrda and Charvat entropy (HCE) [25], Arimoto entropy (AE) [26], Tsallis entropy (TE) [27], extropy (Ex) [28], weighted extropy (WEx) [29] and residual extropy (REx) [30] are computed.
- Eight different methods are implemented to estimate the parameters *β*, *ϑ* and *ζ* of the MKWD. These methods are maximum likelihood estimation (MLE), Cramer-von-Mises estimation (CME), maximum product of spacings estimation (MPSE), least squares estimation (LSE), weighted least squares estimation (WLSE), minimum spacing absolute-log distance estimation (MSALDE), percentile estimation (PE) and minimum spacing square-log distance estimation (MSSLE).
- We develop a quantile regression to analyze the connections between dependent and independent variables, and illustrate the implementation of our models using survival times data.

This study addresses the following four research questions (RQ) and related hypotheses (H) to contribute to the field of reliability analysis and lifetime modeling:

RQ1 Can the MKWD effectively model lifetime scenarios characterized by non-monotonic failure rates, which are inadequately handled by the traditional WD?
H1 The MKWD will provide a more accurate fit for lifetime data with non-monotonic failure rates compared to the existing Weibull variants.
RQ2 What are the statistical properties of the MKWD, and how do these properties enhance its adaptability in different reliability analysis contexts?
H2 The MKWD will exhibit diverse statistical characteristics (e.g., moments, entropy measures) that make it suitable for a wide range of applications in reliability and survival analysis.
RQ3 Which parameter estimation method(s) provide the most reliable estimates for the MKWD parameters in practical applications?
H3 MLE will outperform other estimation methods in terms of accuracy and reliability.
RQ4 How does the MKWD perform in real-world data applications?
H4 The MKWD will demonstrate superior modeling performance when applied to real-world datasets, providing better fits and more accurate predictions than competing distributions.

The subsequent sections of this work are structured in the following manner: the development of the MKWD is described in Section 2. The statistical features of the MKWD are outlined in Section 3. Some measures of entropy and extropy are discussed in Sections 4 and 5, respectively. Section 6 discusses eight approaches to estimate the parameters of the MKWD. Additionally, Monte Carlo simulations are conducted to evaluate the adequacy of these strategies in Section 7. Data analysis using two real data sets is conducted in Section 8. Section 9 presents the formulation of the quantile regression, followed by simulation experiments and applications. The results of the study are summarized in Section 10.

## 2 Formulation of the Modified Kies Weibull distribution

In this section, we construct the MKWD by inserting (1) and (2) into (3) and (4). Then, the MKWD has the following cdf, pdf, reliability function (rf) and hrf:

$$\begin{array}{c} F\left(y;\beta ,\vartheta ,\zeta \right)=1-e^{-{\left[e^{\beta y^{\vartheta }}-1\right]}^{\zeta }},\;\;\;\;\;\;\;y>0,\;\;\beta ,\vartheta ,\zeta >0, \end{array}$$ (7)

$$\begin{array}{c} f\left(y;\beta ,\vartheta ,\zeta \right)=\zeta \beta \vartheta y^{\vartheta -1}e^{\beta \zeta y^{\vartheta }}[1-e^{-\beta y^{\vartheta }}{]}^{\zeta -1}e^{-{\left[e^{\beta y^{\vartheta }}-1\right]}^{\zeta }}, \end{array}$$ (8)

$$R\left(y;\beta ,\vartheta ,\zeta \right)=e^{-{\left[e^{\beta y^{\vartheta }}-1\right]}^{\zeta }},\;\;\;\;\;\;\;y>0,\;\;\beta ,\vartheta ,\zeta >0,$$

and

$$h\left(y;\beta ,\vartheta ,\zeta \right)=\zeta \beta \vartheta y^{\vartheta -1}e^{\beta \zeta y^{\vartheta }}[1-e^{-\beta y^{\vartheta }}{]}^{\zeta -1}.$$

**Fig 1:**
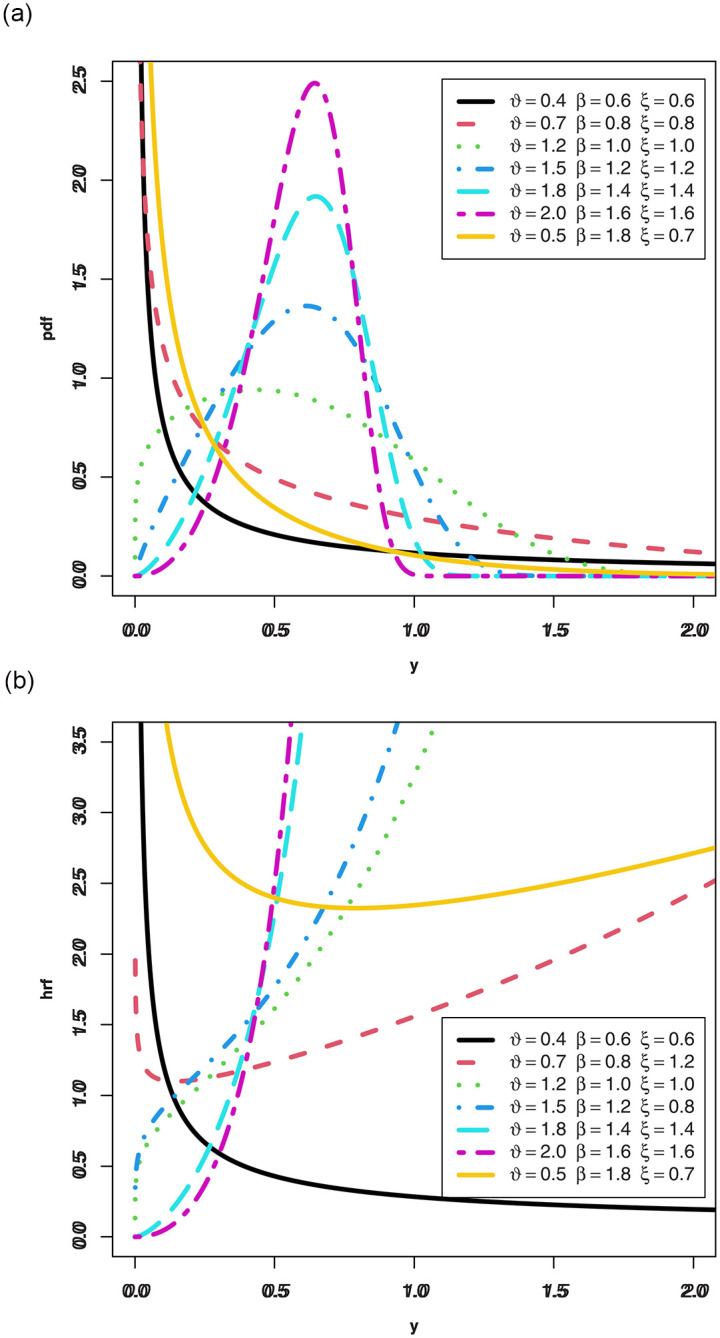
Plots of pdf and hrf for the MKWD.

The reversed hrf (rhrf), cumulative hrf (chrf), odd ratio (OR), failure rate average (FRA) and Mills ratio (MR) of the MKWD are

$$\tau \left(y;\beta ,\vartheta ,\zeta \right)=\frac{\zeta \beta \vartheta y^{\vartheta -1}e^{\beta \zeta y^{\vartheta }}[1-e^{-\beta y^{\vartheta }}{]}^{\zeta -1}e^{-{\left[e^{\beta y^{\vartheta }}-1\right]}^{\zeta }}}{1-e^{-{\left[e^{\beta y^{\vartheta }}-1\right]}^{\zeta }}},$$

$$\text{H}\left(y;\beta ,\vartheta ,\zeta \right)=\frac{-1}{{\left[e^{\beta y^{\vartheta }}-1\right]}^{\zeta }},$$

$$\text{OR}\left(y;\beta ,\vartheta ,\zeta \right)=e^{{\left[e^{\beta y^{\vartheta }}-1\right]}^{\zeta }}-1,$$

$$\text{FRA}\left(y;\beta ,\vartheta ,\zeta \right)=\frac{-1}{y{\left[e^{\beta y^{\vartheta }}-1\right]}^{\zeta }},$$

and

$$\text{MR}\left(y;\beta ,\vartheta ,\zeta \right)=\frac{1}{\zeta \beta \vartheta y^{\vartheta -1}e^{\beta \zeta y^{\vartheta }}[1-e^{-\beta y^{\vartheta }}{]}^{\zeta -1}},$$

respectively. Fig 1 shows that the pdf for the MKWD can have declining, right-skewed and unimodal forms, and the hrf can be bathtub, declining, increasing and *J*-shaped for the MKWD.

## 3 Statistical properties

Regarding the statistical characteristics of the MKWD, we discuss them in this section.

### 3.1 Quantile function

The quantile function is a frequently employed tool in general statistics for determining the mathematical properties of a distribution and percentiles. The quantile function of the MKWD, say *Q*(*u*), 0 < *u* < 1, defined by *F*(*Q*(*u*)) = *u*, can be computed as follows:

$$\begin{array}{c} y_{u}=Q\left(u\right)=[\frac{1}{\beta }\text{log}(1+{\left[\text{log}(\frac{1}{1-u})\right]}^{\frac{1}{\zeta }}){]}^{\frac{1}{\vartheta }}. \end{array}$$ (9)

To investigate the median (*m*) of the MKWD, we set *u* = 0.5 in Eq (9) as follows:

$$m=[\frac{1}{\beta }\text{log}(1+{\left[\text{log}(2)\right]}^{\frac{1}{\zeta }}){]}^{\frac{1}{\vartheta }}.$$

### 3.2 Moments and moment generating function

In statistics, moments of probability distributions are measures related to the structure of the graph. The variance is the second moment around the mean in a probability distribution, whereas the mean value corresponds to the first moment. The ratio of the third mean moment to the variance is the definition of the skewness measure. The ratio of the standard deviation to the fourth power to the fourth moment about the mean is the definition of the kurtosis measure. For any positive integer *r*, the *r*th moment of the MKWD can be determined as follows:

$$\begin{array}{c} \mu_{r}^{\prime }=\int_{0}^{\infty }y^{r}f\left(y;\varphi \right)dy, \end{array}$$ (10)

where *φ* = (*β*, *ϑ*, *ζ*). By inserting (5) into (10), we have

$$\begin{array}{c} \mu_{r}^{\prime }=\beta \vartheta \sum_{i,j=0}^{\infty }\eta_{i,j}\int_{0}^{\infty }y^{r+\vartheta -1}e^{-\beta y^{\vartheta }}{\left(1-e^{-\beta y^{\vartheta }}\right)}^{\zeta (i+1)+j-1}dy. \end{array}$$ (11)

By using the binomial expansion in the last term in (11), we get

$$\begin{array}{c} \mu_{r}^{\prime }=\sum_{i,j,k=0}^{\infty }\eta_{i,j,k}\int_{0}^{\infty }y^{r+\vartheta -1}e^{-\beta \left(k+1\right)y^{\vartheta }}dy, \end{array}$$ (12)

where $\eta_{i,j,k}=\beta \vartheta \eta_{i,j}{\left(-1\right)}^{k}(\begin{array}{c} \zeta (i+1)+j-1\\ k \end{array}).$ Let *z* = *β*(*k* + 1)*y*^*ϑ*^, then

$$\begin{array}{c} \mu_{r}^{\prime }=\sum_{i,j,k=0}^{\infty }\frac{\eta_{i,j,k}}{\vartheta {\left[(k+1)\beta \right]}^{\frac{r}{\vartheta }+1}}\int_{0}^{\infty }z^{\frac{r}{\vartheta }}e^{-z}dz=\sum_{i,j,k=0}^{\infty }\frac{\eta_{i,j,k}\Gamma (\frac{r}{\vartheta }+1)}{\vartheta {\left[(k+1)\beta \right]}^{\frac{r}{\vartheta }+1}}, \end{array}$$ (13)

where Γ(., .) is the gamma function (GFN). The moment generating function of the MKWD is calculated below:

$$M_{Y}\left(t\right)=E\left(e^{tY}\right)=\int_{0}^{\infty }e^{ty}f\;\left(y;\varphi \right)dy=\sum_{r=0}^{\infty }\frac{t^{r}}{r!}\mu_{r}^{\prime }=\sum_{i,j,k=0}^{\infty }\sum_{r=0}^{\infty }\frac{t^{r}}{r!}\frac{\eta_{i,j,k}\Gamma (\frac{r}{\vartheta }+1)}{\vartheta {\left[(k+1)\beta \right]}^{\frac{r}{\vartheta }+1}}.$$

Tables 1 and 2 show the numerical values of the moments $\mu_{1}^{\prime }$, $\mu_{2}^{\prime }$, $\mu_{3}^{\prime }$ and $\mu_{4}^{\prime }$, and the numerical values of the variance (*σ*^2^), coefficient of skewness (CS), coefficient of kurtosis (CK) and coefficient of variation (CV) associated with the MKWD. It can be observed from Tables 1 and 2 that as the parameters *ϑ* and *β* increase while keeping *ζ* constant, there is a general trend of decreasing values in the first four moments and *σ*^2^, and CS, CK, and CV show an decreasing trend. This pattern indicates a significant sensitivity of the distribution to changes in these parameters of the distribution.

**Table 1:** Some numerical values of moments for the MKWD where *ζ* = 0.8.

*ϑ*	*β*	$\mu_{1}^{\prime }$	$\mu_{2}^{\prime }$	$\mu_{3}^{\prime }$	$\mu_{4}^{\prime }$	*σ* ^2^	*σ*	CS	CK	CV
1.5	0.5	0.4433	0.5355	0.8963	1.8066	0.3390	0.5822	1.8161	6.3793	1.3135
	0.6	0.4007	0.3870	0.5136	0.8201	0.2264	0.4758	1.6431	5.7022	1.1875
	0.7	0.3748	0.3041	0.3345	0.4410	0.1637	0.4046	1.4780	5.1016	1.0795
	0.8	0.3582	0.2535	0.2392	0.2689	0.1252	0.3538	1.3254	4.5930	0.9875
	0.9	0.3474	0.2204	0.1833	0.1803	0.0997	0.3158	1.1866	4.1730	0.9090
	1	0.3403	0.1978	0.1480	0.1301	0.0820	0.2864	1.0611	3.8309	0.8416
	1.1	0.3355	0.1816	0.1245	0.0994	0.0691	0.2628	0.9480	3.5544	0.7833
	1.2	0.3324	0.1698	0.1081	0.0795	0.0593	0.2435	0.8458	3.3319	0.7325
	1.3	0.3304	0.1608	0.0961	0.0659	0.0517	0.2273	0.7532	3.1534	0.6879
	1.4	0.3292	0.1540	0.0872	0.0562	0.0456	0.2135	0.6690	3.0106	0.6485
2.1	0.5	0.2911	0.2309	0.2538	0.3359	0.1462	0.3823	1.8161	6.3793	1.3135
	0.6	0.2631	0.1669	0.1454	0.1525	0.0976	0.3125	1.6431	5.7022	1.1875
	0.7	0.2461	0.1311	0.0947	0.0820	0.0706	0.2657	1.4780	5.1016	1.0795
	0.8	0.2352	0.1093	0.0677	0.0500	0.0540	0.2323	1.3254	4.5930	0.9875
	0.9	0.2281	0.0951	0.0519	0.0335	0.0430	0.2074	1.1866	4.1730	0.9090
	1	0.2234	0.0853	0.0419	0.0242	0.0354	0.1880	1.0611	3.8309	0.8416
	1.1	0.2203	0.0783	0.0353	0.0185	0.0298	0.1726	0.9480	3.5544	0.7833
	1.2	0.2183	0.0732	0.0306	0.0148	0.0256	0.1599	0.8457	3.3319	0.7325
	1.3	0.2170	0.0694	0.0272	0.0123	0.0223	0.1493	0.7532	3.1534	0.6879
	1.4	0.2162	0.0664	0.0247	0.0105	0.0197	0.1402	0.6690	3.0106	0.6485
2.5	0.5	0.2341	0.1493	0.1320	0.1405	0.0945	0.3074	1.8161	6.3793	1.3135
	0.6	0.2116	0.1079	0.0756	0.0638	0.0631	0.2513	1.6431	5.7022	1.1875
	0.7	0.1979	0.0848	0.0493	0.0343	0.0456	0.2136	1.4780	5.1016	1.0795
	0.8	0.1892	0.0707	0.0352	0.0209	0.0349	0.1868	1.3254	4.5930	0.9875
	0.9	0.1835	0.0615	0.0270	0.0140	0.0278	0.1668	1.1866	4.1730	0.9090
	1	0.1797	0.0552	0.0218	0.0101	0.0229	0.1512	1.0611	3.8309	0.8416
	1.1	0.1772	0.0507	0.0183	0.0077	0.0193	0.1388	0.9480	3.5544	0.7833
	1.2	0.1755	0.0473	0.0159	0.0062	0.0165	0.1286	0.8457	3.3319	0.7325
	1.3	0.1745	0.0449	0.0142	0.0051	0.0144	0.1200	0.7532	3.1534	0.6879
	1.4	0.1739	0.0429	0.0128	0.0044	0.0127	0.1127	0.6690	3.0106	0.6485
2.8	0.5	0.2032	0.1125	0.0863	0.0797	0.0712	0.2668	1.8161	6.3793	1.3135
	0.6	0.1837	0.0813	0.0494	0.0362	0.0476	0.2181	1.6431	5.7022	1.1875
	0.7	0.1718	0.0639	0.0322	0.0195	0.0344	0.1854	1.4780	5.1016	1.0795
	0.8	0.1642	0.0532	0.0230	0.0119	0.0263	0.1621	1.3254	4.5930	0.9875
	0.9	0.1592	0.0463	0.0176	0.0080	0.0210	0.1447	1.1866	4.1730	0.9090
	1	0.1560	0.0416	0.0143	0.0057	0.0172	0.1313	1.0611	3.8310	0.8416
	1.1	0.1538	0.0382	0.0120	0.0044	0.0145	0.1205	0.9480	3.5544	0.7833
	1.2	0.1523	0.0357	0.0104	0.0035	0.0125	0.1116	0.8457	3.3319	0.7325
	1.3	0.1514	0.0338	0.0093	0.0029	0.0109	0.1042	0.7532	3.1534	0.6879
	1.4	0.1509	0.0324	0.0084	0.0025	0.0096	0.0979	0.6690	3.0106	0.6485

**Table 2:** Some numerical values of moments for the MKWD where *ζ* = 0.6.

*ϑ*	*β*	$\mu_{1}^{\prime }$	$\mu_{2}^{\prime }$	$\mu_{3}^{\prime }$	$\mu_{4}^{\prime }$	*σ* ^2^	*σ*	CS	CK	CV
1.5	0.5	0.4476	0.7363	1.8066	5.5771	0.5359	0.7321	2.5419	10.8177	1.6356
	0.6	0.3764	0.4560	0.8201	1.8563	0.3143	0.5606	2.3376	9.6075	1.4893
	0.7	0.3332	0.3164	0.4410	0.7716	0.2053	0.4531	2.1363	8.4822	1.3598
	0.8	0.3053	0.2383	0.2689	0.3788	0.1450	0.3808	1.9469	7.4951	1.2473
	0.9	0.2865	0.1907	0.1803	0.2114	0.1086	0.3295	1.7730	6.6570	1.1500
	1	0.2734	0.1597	0.1301	0.1303	0.0849	0.2914	1.6152	5.9573	1.0658
	1.1	0.2641	0.1384	0.0994	0.0870	0.0687	0.2621	1.4728	5.3778	0.9927
	1.2	0.2573	0.1233	0.0795	0.0619	0.0571	0.2390	1.3445	4.8992	0.9289
	1.3	0.2523	0.1121	0.0659	0.0464	0.0485	0.2202	1.2287	4.5038	0.8727
	1.4	0.2486	0.1037	0.0562	0.0363	0.0419	0.2046	1.1238	4.1764	0.8231
2.1	0.5	0.2555	0.2399	0.3359	0.5919	0.1746	0.4178	2.5421	10.8181	1.6355
	0.6	0.2149	0.1485	0.1525	0.1970	0.1024	0.3200	2.3376	9.6076	1.4892
	0.7	0.1902	0.1031	0.0820	0.0819	0.0669	0.2586	2.1363	8.4822	1.3598
	0.8	0.1743	0.0776	0.0500	0.0402	0.0473	0.2174	1.9469	7.4951	1.2473
	0.9	0.1635	0.0621	0.0335	0.0224	0.0354	0.1881	1.7730	6.6570	1.1500
	1	0.1561	0.0520	0.0242	0.0138	0.0277	0.1663	1.6152	5.9573	1.0658
	1.1	0.1507	0.0451	0.0185	0.0092	0.0224	0.1496	1.4728	5.3778	0.9927
	1.2	0.1468	0.0402	0.0148	0.0066	0.0186	0.1364	1.3445	4.8992	0.9289
	1.3	0.1440	0.0365	0.0123	0.0049	0.0158	0.1257	1.2287	4.5038	0.8727
	1.4	0.1419	0.0338	0.0105	0.0038	0.0136	0.1168	1.1238	4.1764	0.8231
2.5	0.5	0.1911	0.1341	0.1405	0.1851	0.0976	0.3125	2.5419	10.8177	1.6355
	0.6	0.1607	0.0831	0.0638	0.0616	0.0573	0.2393	2.3376	9.6075	1.4893
	0.7	0.1422	0.0576	0.0343	0.0256	0.0374	0.1934	2.1363	8.4822	1.3598
	0.8	0.1303	0.0434	0.0209	0.0126	0.0264	0.1625	1.9469	7.4951	1.2473
	0.9	0.1223	0.0347	0.0140	0.0070	0.0198	0.1406	1.7730	6.6570	1.1500
	1	0.1167	0.0291	0.0101	0.0043	0.0155	0.1244	1.6152	5.9573	1.0658
	1.1	0.1127	0.0252	0.0077	0.0029	0.0125	0.1119	1.4728	5.3778	0.9927
	1.2	0.1098	0.0225	0.0062	0.0021	0.0104	0.1020	1.3445	4.8992	0.9289
	1.3	0.1077	0.0204	0.0051	0.0015	0.0088	0.0940	1.2287	4.5038	0.8727
	1.4	0.1061	0.0189	0.0044	0.0012	0.0076	0.0873	1.1238	4.2329	0.8231
2.8	0.5	0.1582	0.0919	0.0797	0.0870	0.0669	0.2587	2.5419	10.8177	1.6355
	0.6	0.1330	0.0569	0.0362	0.0289	0.0392	0.1981	2.3376	9.6075	1.4893
	0.7	0.1178	0.0395	0.0195	0.0120	0.0256	0.1601	2.1363	8.4822	1.3598
	0.8	0.1079	0.0298	0.0119	0.0059	0.0181	0.1346	1.9469	7.4951	1.2473
	0.9	0.1012	0.0238	0.0080	0.0033	0.0136	0.1164	1.7730	6.6570	1.1500
	1	0.0966	0.0199	0.0057	0.0020	0.0106	0.1030	1.6152	5.9573	1.0658
	1.1	0.0933	0.0173	0.0044	0.0014	0.0086	0.0926	1.4728	5.3778	0.9927
	1.2	0.0909	0.0154	0.0035	0.0010	0.0071	0.0844	1.3445	4.8992	0.9289
	1.3	0.0892	0.0140	0.0029	0.0007	0.0061	0.0778	1.2287	4.5039	0.8727
	1.4	0.0879	0.0130	0.0025	0.0006	0.0052	0.0723	1.1238	4.1764	0.8231

### 3.3 Incomplete and conditional moments

Incomplete moments are often used to assess inequalities, such as income quantiles, BON, LOR and ZEN curves. The *p*th incomplete moment of the MKWD is computed as follows:

$$\begin{array}{c} \xi_{p}\left(t\right)=\int_{0}^{t}y^{p}f\left(y;\varphi \right)dy=\sum_{i,j,k=0}^{\infty }\frac{\eta_{i,j,k}\gamma (\frac{p}{\vartheta }+1,\beta \left(k+1\right)t^{\vartheta })}{\vartheta {\left[(k+1)\beta \right]}^{\frac{p}{\vartheta }+1}}, \end{array}$$ (14)

where $\gamma \left(s,t\right)=\int_{0}^{t}y^{s-1}e^{-y}dy$ is the lower incomplete GFN. Conditional moments are essential in many statistical approaches, such as regression and hypothesis testing, since they demonstrate the relationship between variables and their responses in different circumstances. For example, in regression analysis, these moments can predict the dependent variable’s value based on certain independent variable values, providing significant insights into the variable’s behavior under various situations and improving prediction accuracy. The *p*th conditional moment of the MKWD is computed as follows:

$$\begin{array}{c} \Omega_{p}\left(t\right)=\int_{t}^{\infty }y^{p}f\left(y;\varphi \right)dy=\sum_{i,j,k=0}^{\infty }\frac{\eta_{i,j,k}\Gamma (\frac{r}{\vartheta }+1,\beta \left(k+1\right)t^{\vartheta })}{\vartheta {\left[(k+1)\beta \right]}^{\frac{r}{\vartheta }+1}}, \end{array}$$ (15)

where $\Gamma \left(s,t\right)=\int_{t}^{\infty }y^{s-1}e^{-y}dy$ is the upper incomplete GFN.

### 3.4 Inequality measures

Our primary focus in this subsection is on the LOR, BON, and ZEN curves, which are helpful in demography, econometrics, medicine, survival analysis, and insurance applications. For the MKWD, the LOR, BON, and ZEN curves are provided by

$$\text{LOR}=\frac{\int_{0}^{t}yf\left(y;\varphi \right)dy}{E(Y)}=\frac{\sum_{i,j,k=0}^{\infty }\eta_{i,j,k}\gamma (\frac{1}{\vartheta }+1,\beta \left(k+1\right)t^{\vartheta })}{\sum_{i,j,k=0}^{\infty }\eta_{i,j,k}\Gamma (\frac{1}{\vartheta }+1)},$$

$$\text{BON}=\frac{\text{LOR}}{F(t)}=\frac{\sum_{i,j,k=0}^{\infty }\eta_{i,j,k}\gamma (\frac{1}{\vartheta }+1,\beta \left(k+1\right)t^{\vartheta })}{\left(1-e^{-{\left[e^{\beta t^{\vartheta }}-1\right]}^{\zeta }})\sum_{i,j,k=0}^{\infty }\eta_{i,j,k}\Gamma (\frac{1}{\vartheta }+1\right)},$$

and

$$\text{ZEN}=1-\frac{(\text{LOR})R\left(t\right)}{\int_{t}^{\infty }yf\left(y;\varphi \right)dy}=1-\frac{e^{-{\left[e^{\beta t^{\vartheta }}-1\right]}^{\zeta }}[\sum_{i,j,k=0}^{\infty }\eta_{i,j,k}\gamma (\frac{1}{\vartheta }+1,\beta \left(k+1\right)t^{\vartheta })]}{\left[\sum_{i,j,k=0}^{\infty }\eta_{i,j,k}\Gamma (\frac{1}{\vartheta }+1)][\sum_{i,j,k=0}^{\infty }\eta_{i,j,k}\Gamma (\frac{1}{\vartheta }+1,\beta \left(k+1\right)t^{\vartheta })\right]},$$

respectively, where *F*(*t*) and *R*(*t*) are the cdf and rf of the MKWD at time *t*.

### 3.5 Order statistics

Assume that *Y*_1_, *Y*_2_, …, *Y*_*n*_ are *n* random samples from the MKWD with pdf (8) and cdf (7). Suppose that *Y*_(1)_, *Y*_(2)_, …, *Y*_(*n*)_ are the corresponding OS. The pdf of the *q*th OS is provided as follows:

$$\begin{array}{c} f_{Y_{\left(q\right)}}\left(y\right)=\frac{n!}{(q-1)!(n-q)!}f\left(y;\varphi \right)[F(y;\varphi ){]}^{q-1}[1-F(y;\varphi ){]}^{n-q}. \end{array}$$ (16)

By employing (7) and (8) in (16), we obtain the pdf of *Y*_(*q*)_ of OS for the MKWD below:

$$\begin{array}{c} f_{Y_{\left(q\right)}}\left(y\right)=\frac{\zeta \beta \vartheta n!}{(q-1)!(n-q)!}y^{\vartheta -1}e^{\beta \zeta y^{\vartheta }}[1-e^{-\beta y^{\vartheta }}{]}^{\zeta -1}e^{-(n-q+1){\left[e^{\beta y^{\vartheta }}-1\right]}^{\zeta }}(1-e^{-{\left[e^{\beta y^{\vartheta }}-1\right]}^{\zeta }}{)}^{q-1}. \end{array}$$ (17)

By setting *q* = 1 and *n* in (17), we have the lowest OS and the largest OS for the MKWD as follows:

$$f_{Y_{\left(1\right)}}\left(y\right)=\zeta \beta \vartheta ny^{\vartheta -1}e^{\beta \zeta y^{\vartheta }}[1-e^{-\beta y^{\vartheta }}{]}^{\zeta -1}e^{-n{\left[e^{\beta y^{\vartheta }}-1\right]}^{\zeta }},$$

and

$$f_{Y_{\left(n\right)}}\left(y\right)=\zeta \beta \vartheta ny^{\vartheta -1}e^{\beta \zeta y^{\vartheta }}[1-e^{-\beta y^{\vartheta }}{]}^{\zeta -1}e^{-{\left[e^{\beta y^{\vartheta }}-1\right]}^{\zeta }}(1-e^{-{\left[e^{\beta y^{\vartheta }}-1\right]}^{\zeta }}{)}^{n-1}.$$

## 4 Entropy measures

In this section, five different entropy measures for the MKWD, namely, the RE, EE, HCE, AE and TE are computed. Table 3 reports some numerical values of the MKWD’s entropy measures.

**Table 3:** Some numerical values of the entropy measures of the MKWD.

*ζ*	*ϑ*	*β*	∇ = 0.5					∇ = 0.7				
			RE	EE	HCE	AE	TE	RE	EE	HCE	AE	TE
0.4	0.1	0.10	1.7216	52.6766	15.1078	51.6766	12.5157	0.3174	2.0768	1.0605	0.8582	0.8171
		0.15	1.7740	59.4250	16.1964	58.4250	13.4175	0.4396	2.7515	1.5349	1.2672	1.1826
		0.20	1.8116	64.7998	17.0198	63.7998	14.0997	0.5272	3.3663	1.9005	1.5922	1.4643
		0.25	1.8412	69.3667	17.6930	68.3667	14.6573	0.5958	3.9428	2.2029	1.8673	1.6973
		0.30	1.8656	73.3764	18.2660	72.3764	15.1320	0.6525	4.4921	2.4634	2.1089	1.8980
		0.35	1.8863	76.9586	18.7647	75.9586	15.5452	0.7006	5.0183	2.6929	2.3249	2.0748
		0.40	1.9041	80.1846	19.2041	79.1846	15.9092	0.7421	5.5226	2.8974	2.5200	2.2324
		0.45	1.9196	83.0952	19.5929	82.0952	16.2313	0.7784	6.0041	3.0808	2.6970	2.3737
		0.50	1.9331	85.7136	19.9370	84.7136	16.5163	0.8103	6.4606	3.2455	2.8575	2.5006
		0.55	1.9447	88.0520	20.2398	87.0520	16.7672	0.8382	6.8896	3.3929	3.0025	2.6142
	0.3	0.10	2.3306	214.0652	32.9081	213.0652	27.2619	1.6734	47.1386	9.4182	9.8324	7.2566
		0.15	2.3830	241.5235	35.1051	240.5235	29.0821	1.7965	62.5894	10.6384	11.4041	8.1967
		0.20	2.4148	259.8859	36.5053	258.8859	30.2420	1.8748	74.9568	11.4702	12.5078	8.8376
		0.25	2.4308	269.6488	37.2296	268.6488	30.8420	1.9208	83.3239	11.9797	13.1964	9.2301
		0.30	2.4326	270.7433	37.3099	269.7433	30.9086	1.9390	86.8912	12.1861	13.4779	9.3891
		0.35	2.4206	263.3673	36.7651	262.3673	30.4572	1.9325	85.6094	12.1126	13.3775	9.3325
		0.40	2.3950	248.3354	35.6306	247.3354	29.5173	1.9022	79.8305	11.7715	12.9139	9.0697
		0.45	2.3562	227.1130	33.9686	226.1130	28.1405	1.8510	70.9570	11.2124	12.1631	8.6390
		0.50	2.3047	201.7043	31.8731	200.7043	26.4045	1.7820	60.5288	10.4888	11.2084	8.0814
		0.55	2.2416	174.4043	29.4684	173.4043	24.4124	1.6989	49.9937	9.6628	10.1429	7.4450
0.8	0.1	0.10	1.8323	67.9681	17.4892	66.9681	14.4886	0.5276	3.3695	1.9023	1.5938	1.4657
		0.15	1.9576	90.6879	20.5764	89.6879	17.0460	0.8223	6.6424	3.3088	2.9196	2.5494
		0.20	2.0414	109.9962	22.9058	108.9962	18.9758	1.0210	10.4952	4.4319	4.0573	3.4147
		0.25	2.1014	126.3064	24.7182	125.3064	20.4772	1.1649	14.6185	5.3473	5.0325	4.1200
		0.30	2.1454	139.7582	26.1265	138.7582	21.6439	1.2721	18.7125	6.0910	5.8546	4.6930
		0.35	2.1772	150.3861	27.1918	149.3861	22.5264	1.3521	22.4955	6.6826	6.5269	5.1489
		0.40	2.1992	158.1918	27.9504	157.1918	23.1549	1.4102	25.7184	7.1338	7.0502	5.4965
		0.45	2.2127	163.1840	28.4258	162.1840	23.5487	1.4499	28.1800	7.4524	7.4251	5.7420
		0.50	2.2185	165.4029	28.6348	164.4029	23.7218	1.4734	29.7421	7.6446	7.6534	5.8901
		0.55	2.2173	164.9354	28.5909	163.9354	23.6854	1.4820	30.3383	7.7161	7.7387	5.9451
	0.3	0.10	2.5902	389.2314	45.2157	388.2314	37.4579	2.2035	159.7710	15.4966	18.1940	11.9398
		0.15	2.6426	439.1029	48.1751	438.1029	39.9096	2.3583	228.1911	17.7337	21.5820	13.6635
		0.20	2.6175	414.4819	46.7364	413.4819	38.7177	2.3576	227.8464	17.7237	21.5665	13.6558
		0.25	2.5302	339.0345	42.0384	338.0345	34.8258	2.2502	177.9112	16.1465	19.1623	12.4406
		0.30	2.3935	247.4611	35.5635	246.4611	29.4618	2.0776	119.5690	13.8457	15.7961	10.6678
		0.35	2.2258	168.1851	28.8948	167.1851	23.9372	1.8820	76.2086	11.5489	12.6135	8.8982
		0.40	2.0497	112.1128	23.1483	111.1128	19.1767	1.6938	49.4035	9.6131	10.0796	7.4067
		0.45	1.8824	76.2741	18.6703	75.2741	15.4670	1.5238	33.4003	8.0686	8.1624	6.2167
		0.50	1.7302	53.7227	15.2809	52.7227	12.6592	1.3719	23.5461	6.8344	6.7020	5.2658
		0.55	1.5922	39.1016	12.6822	38.1016	10.5063	1.2337	17.1257	5.8177	5.5495	4.4825

### 4.1 Rényi entropy

The RE of the MKWD can be computed using the following formula

$$\begin{array}{c} R_{\nabla }\left(\varphi \right)=\frac{1}{1-\nabla }\text{log}[\int_{0}^{\infty }f{\left(y;\varphi \right)}^{\nabla }dy].\nabla >0,\nabla \neq 1. \end{array}$$ (18)

Now, we want to compute the integral $\int_{0}^{\infty }f{\left(y;\varphi \right)}^{\nabla }dy$. By inserting (1) and (2) into (6), we get

$$\begin{array}{c} \int_{0}^{\infty }f{\left(y;\varphi \right)}^{\nabla }dy=(\beta \vartheta {)}^{\nabla }\sum_{i,j=0}^{\infty }{℘}_{i,j}\int_{0}^{\infty }y^{\nabla \vartheta -\nabla }e^{-\nabla \beta y^{\vartheta }}{\left(1-e^{-\beta y^{\vartheta }}\right)}^{\nabla (\zeta -1)+\zeta i+j}dy. \end{array}$$ (19)

By using the binomial expansion for the above Eq (19), we obtain

$$\begin{array}{c} \int_{0}^{\infty }f{\left(y;\varphi \right)}^{\nabla }dy=\sum_{i,j,k=0}^{\infty }{℘}_{i,j,k}\int_{0}^{\infty }y^{\nabla \vartheta -\nabla }e^{-(k+\nabla )\beta y^{\vartheta }}dy, \end{array}$$ (20)

where ${℘}_{i,j,k}=(\beta \vartheta {)}^{\nabla }{℘}_{i,j}(\begin{array}{c} \nabla (\zeta -1)+\zeta i+j\\ k \end{array}).$ Let *z* = (*k* + ∇) *βy*^*ϑ*^, then we have

$$\begin{array}{c} \int_{0}^{\infty }f{\left(y;\varphi \right)}^{\nabla }dy=\sum_{i,j,k=0}^{\infty }\frac{{℘}_{i,j,k}}{\vartheta {\left[(k+\nabla )\beta \right]}^{\nabla -\frac{\nabla }{\vartheta }+\frac{1}{\vartheta }}}\int_{0}^{\infty }z^{\nabla -\frac{\nabla }{\vartheta }+\frac{1}{\vartheta }-1}e^{-z}dz, \end{array}$$ (21)

and the integral $\int_{0}^{\infty }f{\left(y;\varphi \right)}^{\nabla }dy$ can be formulated as

$$\begin{array}{c} \int_{0}^{\infty }f{\left(y;\varphi \right)}^{\nabla }dy=\sum_{i,j,k=0}^{\infty }\frac{{℘}_{i,j,k}\Gamma (\nabla -\frac{\nabla }{\vartheta }+\frac{1}{\vartheta })}{\vartheta {\left[(k+\nabla )\beta \right]}^{\nabla -\frac{\nabla }{\vartheta }+\frac{1}{\vartheta }}}. \end{array}$$ (22)

By employing (22) in (18), the RE of the MKWD is given by

$$\begin{array}{c} R_{\nabla }\left(\varphi \right)=\frac{1}{1-\nabla }\text{log}[\sum_{i,j,k=0}^{\infty }\frac{{℘}_{i,j,k}\Gamma (\nabla -\frac{\nabla }{\vartheta }+\frac{1}{\vartheta })}{\vartheta {\left[(k+\nabla )\beta \right]}^{\nabla -\frac{\nabla }{\vartheta }+\frac{1}{\vartheta }}}].\nabla >0,\nabla \neq 1. \end{array}$$ (23)

### 4.2 Exponential entropy

The EE of the MKWD can be computed using the following formula

$$\begin{array}{c} E_{\nabla }\left(\varphi \right)={\left[\int_{0}^{\infty }f{\left(y;\varphi \right)}^{\nabla }dy\right]}^{\frac{1}{1-\nabla }}. \end{array}$$ (24)

By utilizing (22) in (24), the EE of the MKWD is given by

$$\begin{array}{c} E_{\nabla }\left(\varphi \right)={\left[\sum_{i,j,k=0}^{\infty }\frac{{℘}_{i,j,k}\Gamma (\nabla -\frac{\nabla }{\vartheta }+\frac{1}{\vartheta })}{\vartheta {\left[(k+\nabla )\beta \right]}^{\nabla -\frac{\nabla }{\vartheta }+\frac{1}{\vartheta }}}\right]}^{\frac{1}{1-\nabla }}. \end{array}$$ (25)

### 4.3 Havrda and Charvat entropy

The HCE of the MKWD can be computed using the formula

$$\begin{array}{c} {\text{HC}}_{\nabla }\left(\varphi \right)=\frac{1}{2^{1-\nabla }-1}\left[\int_{0}^{\infty }f{\left(y;\varphi \right)}^{\nabla }dy-1\right]. \end{array}$$ (26)

By inserting (22) into (26), the HCE of the MKWD is given by

$$\begin{array}{c} {\text{HC}}_{\nabla }\left(\varphi \right)=\frac{1}{2^{1-\nabla }-1}\left[\sum_{i,j,k=0}^{\infty }\frac{{℘}_{i,j,k}\Gamma (\nabla -\frac{\nabla }{\vartheta }+\frac{1}{\vartheta })}{\vartheta {\left[(k+\nabla )\beta \right]}^{\nabla -\frac{\nabla }{\vartheta }+\frac{1}{\vartheta }}}-1\right]. \end{array}$$ (27)

### 4.4 Arimoto entropy

The AE of the MKWD can be computed using

$$\begin{array}{c} A_{\nabla }\left(\varphi \right)=\frac{\nabla }{1-\nabla }\{{\left[\int_{0}^{\infty }f{\left(y;\varphi \right)}^{\nabla }dy\right]}^{\frac{1}{\nabla }}-1\}, \end{array}$$ (28)

and by employing (22) in (28), the AE of the MKWD is given by

$$\begin{array}{c} A_{\nabla }\left(\varphi \right)=\frac{\nabla }{1-\nabla }\{{\left[\sum_{i,j,k=0}^{\infty }\frac{{℘}_{i,j,k}\Gamma (\nabla -\frac{\nabla }{\vartheta }+\frac{1}{\vartheta })}{\vartheta {\left[(k+\nabla )\beta \right]}^{\nabla -\frac{\nabla }{\vartheta }+\frac{1}{\vartheta }}}\right]}^{\frac{1}{\nabla }}-1\}. \end{array}$$ (29)

### 4.5 Tsallis entropy

The TE of the MKWD can be computed using the formula

$$\begin{array}{c} T_{\nabla }\left(\varphi \right)=\frac{1}{\nabla -1}\left[1-\int_{0}^{\infty }f{\left(y;\varphi \right)}^{\nabla }dy\right]. \end{array}$$ (30)

Then, by using (22) in (30), the TE of the MKWD is given by

$$\begin{array}{c} T_{\nabla }\left(\varphi \right)=\frac{1}{\nabla -1}\left[1-\sum_{i,j,k=0}^{\infty }\frac{{℘}_{i,j,k}\Gamma (\nabla -\frac{\nabla }{\vartheta }+\frac{1}{\vartheta })}{\vartheta {\left[(k+\nabla )\beta \right]}^{\nabla -\frac{\nabla }{\vartheta }+\frac{1}{\vartheta }}}\right]. \end{array}$$ (31)

In general, Table 3 shows a clear trend where values increase as *β* rises for both values of ∇. Specifically, the measures RE and EE increase with higher values of *β* and ∇, while HCE and AE exhibit a similar upward trend, though at a slower rate.

## 5 Extropy measures

Lad et al. [28] launched Ex in 2015, a new measure of uncertainty. The total log scoring system can be implemented to statistically score forecasting distributions utilizing Ex. For a non-negative random variable *Y*, the Ex is defined as follows:

$$\begin{array}{c} \Phi \left(\varphi \right)=\frac{-1}{2}\int_{0}^{\infty }f^{2}\left(y;\varphi \right)dy\text{.} \end{array}$$ (32)

By setting ∇ = 2 in (22) and inserting into (32), the Ex of the MKW is given by:

$$\begin{array}{c} \Phi \left(\varphi \right)=\frac{-1}{2}\left[\sum_{i,j,k=0}^{\infty }\frac{{℘}_{i,j,k}\Gamma (2-\frac{1}{\vartheta })}{\vartheta {\left[(k+2)\beta \right]}^{2-\frac{1}{\vartheta }}}\right],\;\;\;2\vartheta >1. \end{array}$$ (33)

The concept of the WEx was introduced in [29] and is defined as follows:

$$\begin{array}{c} \Phi^{w}\left(\varphi \right)=\frac{-1}{2}\int_{0}^{\infty }yf^{2}\left(y;\varphi \right)dy\text{.} \end{array}$$ (34)

By setting ∇ = 2 in (22) and substituting the respective expression in (34), the WEx of the MKW is given by:

$$\begin{array}{c} \Phi^{w}\left(\varphi \right)=\frac{-1}{2}\left[\sum_{i,j,k=0}^{\infty }\frac{{℘}_{i,j,k}}{\vartheta {\left[(k+2)\beta \right]}^{2}}\right]. \end{array}$$ (35)

Qiu & Jia [30] defined the extropy for residual lifetime *Y*_*t*_ as the REx at time *t* as:

$$\begin{array}{c} \Phi \left(Y_{t}\right)=\frac{-1}{2{\bar{F}}^{2}\left(t\right)}\int_{t}^{\infty }f^{2}\left(y;\varphi \right)dy\text{.} \end{array}$$ (36)

The REx of the MKWD can thus be expressed as

$$\Phi \left(Y_{t}\right)=\frac{-1}{2{\bar{F}}^{2}\left(t\right)}\left[\sum_{i,j,k=0}^{\infty }\frac{{℘}_{i,j,k}\Gamma (2-\frac{1}{\vartheta },(k+\nabla )\beta t^{\vartheta })}{\vartheta {\left[(k+2)\beta \right]}^{2-\frac{1}{\vartheta }}}\right],\;\;\;2\vartheta >1,$$

where Γ(., *y*) is the upper incomplete GF.

Table 4 shows a few numerical values that are associated with extropy metrics of the MKWD. Overall, Table 4 provides a comprehensive view of how extropy measures vary with different parameter values. The trends suggest that increasing values of *ζ* and *ϑ* generally lead to more negative extropy values, indicating increased extropy as these parameters change.

**Table 4:** Some numerical values of the entropy measures of the MKWD (*β* = 0.2).

*ζ*	*ϑ*	Ex	WEx	REx		
				*t* = 0.5	*t* = 1.2	*t* = 1.8
0.5	1.1	-0.299	-0.097	-0.070	-0.066	-0.067
	1.2	-0.183	-0.106	-0.081	-0.079	-0.082
	1.3	-0.149	-0.115	-0.091	-0.093	-0.098
	1.4	-0.137	-0.124	-0.102	-0.107	-0.116
	1.5	-0.132	-0.133	-0.112	-0.122	-0.135
	1.6	-0.131	-0.141	-0.123	-0.137	-0.157
	1.7	-0.133	-0.150	-0.133	-0.153	-0.180
0.7	1.1	-0.091	-0.133	-0.082	-0.086	-0.091
	1.2	-0.095	-0.145	-0.094	-0.102	-0.111
	1.3	-0.101	-0.157	-0.106	-0.119	-0.133
	1.4	-0.109	-0.169	-0.118	-0.136	-0.157
	1.5	-0.117	-0.181	-0.130	-0.155	-0.183
	1.6	-0.125	-0.193	-0.143	-0.174	-0.213
	1.7	-0.134	-0.205	-0.155	-0.194	-0.245
0.9	1.1	-0.084	-0.169	-0.090	-0.101	-0.111
	1.2	-0.094	-0.184	-0.104	-0.120	-0.136
	1.3	-0.105	-0.200	-0.118	-0.139	-0.163
	1.4	-0.117	-0.215	-0.131	-0.160	-0.194
	1.5	-0.129	-0.230	-0.145	-0.182	-0.227
	1.6	-0.141	-0.246	-0.159	-0.204	-0.265
	1.7	-0.154	-0.261	-0.173	-0.227	-0.306
1.2	1.1	-0.094	-0.224	-0.102	-0.118	-0.137
	1.2	-0.109	-0.245	-0.118	-0.141	-0.168
	1.3	-0.124	-0.265	-0.134	-0.164	-0.203
	1.4	-0.140	-0.285	-0.151	-0.188	-0.242
	1.5	-0.156	-0.306	-0.168	-0.214	-0.286
	1.6	-0.173	-0.326	-0.185	-0.240	-0.335
	1.7	-0.190	-0.346	-0.203	-0.268	-0.390
1.4	1.1	-0.103	-0.261	-0.110	-0.128	-0.151
	1.2	-0.121	-0.285	-0.128	-0.152	-0.186
	1.3	-0.139	-0.309	-0.147	-0.178	-0.225
	1.4	-0.158	-0.333	-0.166	-0.204	-0.270
	1.5	-0.177	-0.357	-0.185	-0.232	-0.320
	1.6	-0.196	-0.380	-0.205	-0.261	-0.377
	1.7	-0.216	-0.404	-0.225	-0.290	-0.442

## 6 Estimation methods

In this section, we implement eight approaches to estimate the parameters *β*, *ϑ* and *ζ* of the MKWD. These methods are MLE, CME, MPSE, LSE, WLSE, MSALDE, PE, and MSSLE.

### 6.1 Maximum likelihood method

The maximum likelihood method [31, 32] is based on maximizing the (log-)likelihood function to estimate the parameters. This method is one of the most widely used estimation techniques, providing estimators with desirable properties such as consistency and asymptotic efficiency. The log-likelihood function is given by

$$\begin{array}{c} l=n\;\text{log}\left(\zeta \right)+n\;\text{log}\left(\beta \right)+n\;\text{log}\left(\vartheta \right)+\left(\vartheta -1\right)\sum_{i=1}^{n}\text{log}\left(y_{i}\right)+\beta \zeta \sum_{i=1}^{n}y_{i}^{\vartheta }+\left(\zeta -1\right)\sum_{i=1}^{n}\text{log}\left[1-e^{-\beta y_{i}^{\vartheta }}\right]-\sum_{i=1}^{n}{\left[e^{\beta y_{i}^{\vartheta }}-1\right]}^{\zeta }, \end{array}$$

and the partial derivatives of *l* are as follows:

$$\begin{array}{c} \frac{\partial l}{\partial \beta }=\frac{n}{\beta }+\zeta \sum_{i=1}^{n}y_{i}^{\vartheta }+\left(\zeta -1\right)\sum_{i=1}^{n}\frac{y_{i}^{\vartheta }e^{-\beta y_{i}^{\vartheta }}}{1-e^{-\beta y_{i}^{\vartheta }}}-\zeta \sum_{i=`}^{n}y_{i}^{\vartheta }e^{\beta y_{i}^{\vartheta }}{\left[e^{\beta y_{i}^{\vartheta }}-1\right]}^{\zeta -1},\\ \frac{\partial l}{\partial \vartheta }=\frac{n}{\vartheta }+\sum_{i=1}^{n}\text{log}\left(y_{i}\right)+\beta \zeta \sum_{i=1}^{n}y_{i}^{\vartheta }\;\text{log}\left(y_{i}\right)+\beta \left(\zeta -1\right)\sum_{i=1}^{n}\frac{y_{i}^{\vartheta }e^{-\beta y_{i}^{\vartheta }}\text{log}\left(y_{i}\right)}{1-e^{-\beta y_{i}^{\vartheta }}}-\zeta \beta \sum_{i=`}^{n}y_{i}^{\vartheta }e^{\beta y_{i}^{\vartheta }}\text{log}\left(y_{i}\right){\left[e^{\beta y_{i}^{\vartheta }}-1\right]}^{\zeta -1},\\ \frac{\partial l}{\partial \zeta }=\frac{n}{\zeta }+\beta \sum_{i=1}^{n}y_{i}^{\vartheta }+\sum_{i=1}^{n}\text{log}\left[1-e^{-\beta y_{i}^{\vartheta }}\right]-\sum_{i=1}^{n}{\left[e^{\beta y_{i}^{\vartheta }}-1\right]}^{\zeta }\text{log}\left[e^{\beta y_{i}^{\vartheta }}-1\right]. \end{array}$$

To estimate the parameters *β*, *ϑ* and *ζ* it is required to solve the system of equations $\frac{\partial l}{\partial \beta }=0$, $\frac{\partial l}{\partial \vartheta }=0$ and $\frac{\partial l}{\partial \zeta }=0$, which has no closed form and therefore is to be solved numerically.

### 6.2 Cramér-von-Mises method

The Cramér-von-Mises method [33] minimizes the distance between the empirical and theoretical cumulative distribution functions. It is useful when the goal is to achieve a good fit across the entire range of the data, not just the tails. CME depends on minimizing the function

$$C\left(\beta ,\vartheta ,\zeta \right)=\frac{1}{12n}+\sum_{i=1}^{n}{\left[F\left(y_{\left(i\right)}\right)-\frac{2i-1}{2n}\right]}^{2}.$$

From now on we use the following notations:

$$\begin{array}{c} F_{i}=F\left(y_{\left(i\right)};\beta ,\vartheta ,\zeta \right), \end{array}$$ (37)

$$\begin{array}{c} F_{\beta_{i}}=\zeta y_{\left(i\right)}^{\vartheta }e^{\beta y_{\left(i\right)}^{\vartheta }}{\left[e^{\beta y_{\left(i\right)}^{\vartheta }}-1\right]}^{\zeta -1}e^{-{\left[e^{\beta y_{\left(i\right)}^{\vartheta }}-1\right]}^{\zeta }}, \end{array}$$ (38)

$$\begin{array}{c} F_{\vartheta_{i}}=\zeta \beta y_{\left(i\right)}^{\vartheta }\;\text{log}\left(y_{\left(i\right)}\right)e^{\beta y_{\left(i\right)}^{\vartheta }}{\left[e^{\beta y_{\left(i\right)}^{\vartheta }}-1\right]}^{\zeta -1}e^{-{\left[e^{\beta y_{\left(i\right)}^{\vartheta }}-1\right]}^{\zeta }}, \end{array}$$ (39)

and

$$\begin{array}{c} F_{\zeta_{i}}={\left[e^{\beta y_{\left(i\right)}^{\vartheta }}-1\right]}^{\zeta }\;\text{log}\left[e^{\beta y_{\left(i\right)}^{\vartheta }}-1\right]e^{-{\left[e^{\beta y_{\left(i\right)}^{\vartheta }}-1\right]}^{\zeta }}. \end{array}$$ (40)

The aim is to compute the partial derivatives of *C*(*β*, *ϑ*, *ζ*) with respect to *β*, *ϑ* and *ζ*, by using Eqs (37), (38), (39) and (40),

$$\frac{\partial C(\beta ,\vartheta ,\zeta )}{\partial \beta }=2\sum_{i=1}^{n}F_{\beta_{i}}\left[F_{i}-\frac{2i-1}{2n}\right],$$

$$\frac{\partial C(\beta ,\vartheta ,\zeta )}{\partial \vartheta }=2\sum_{i=1}^{n}F_{\vartheta_{i}},\left[F_{i}-\frac{2i-1}{2n}\right],$$

$$\frac{\partial C(\beta ,\vartheta ,\zeta )}{\partial \zeta }=2\sum_{i=1}^{n}F_{\zeta_{i}}\left[F_{i}-\frac{2i-1}{2n}\right].$$

Then the system of equations $\frac{\partial C(\beta ,\vartheta ,\zeta )}{\partial \beta }=0$, $\frac{\partial C(\beta ,\vartheta ,\zeta )}{\partial \vartheta }=0$ and $\frac{\partial C(\beta ,\vartheta ,\zeta )}{\partial \zeta }=0$ is solved using numerical methods.

### 6.3 Maximum product of spacings method

MPSE is known for providing efficient estimates by maximizing the spacing between ordered statistics. It is particularly useful in continuous distributions. This method needs to maximize the MPS function to estimate the parameters *β*, *ϑ* and *ζ* [34],

$$\delta (\beta ,\vartheta ,\zeta )=\frac{1}{n+1}\sum_{i=1}^{n+1}\;\text{log}\Lambda_{i}\left(y_{\left(i\right)}\right),$$

where *Λ*_*i*_(*y*_(*i*)_) = *F*(*y*_(*i*)_) − *F*(*y*_(*i*−1)_), *F*(*y*_(0)_) = 0 and *F*(*y*_(*n*+1)_) = 1. Using Eqs (37), (38), (39) and (40), the partial derivatives with respect to *β*, *ϑ* and *ζ* are given by

$$\frac{\partial \delta (\beta ,\vartheta ,\zeta )}{\partial \beta }=\frac{1}{n+1}\sum_{i=1}^{n+1}\frac{F_{\beta_{i}}-F_{\beta_{i-1}}}{F_{i}-F_{i-1}},$$

$$\frac{\partial \delta (\beta ,\vartheta ,\zeta )}{\partial \vartheta }=\frac{1}{n+1}\sum_{i=1}^{n+1}\frac{F_{\vartheta_{i}}-F_{\vartheta_{i-1}}}{F_{i}-F_{i-1}},$$

$$\frac{\partial \delta (\beta ,\vartheta ,\zeta )}{\partial \zeta }=\frac{1}{n+1}\sum_{i=1}^{n+1}\frac{F_{\zeta_{i}}-F_{\zeta_{i-1}}}{F_{i}-F_{i-1}}.$$

Through solving the equations $\frac{\partial \delta (\beta ,\vartheta ,\zeta )}{\partial \beta }=0$, $\frac{\partial \delta (\beta ,\vartheta ,\zeta )}{\partial \vartheta }=0$ and $\frac{\partial \delta (\beta ,\vartheta ,\zeta )}{\partial \zeta }=0$, we get the estimates of *β*, *ϑ* and *ζ*.

### 6.4 Least squares method

LSE is selected for its straightforward approach, minimizing the squared differences between observed and theoretical quantiles. The LSE depends on minimizing the function [35]

$$V\left(\beta ,\vartheta ,\zeta \right)=\sum_{i=1}^{n}{\left[F\left(y_{\left(i\right)}\right)-\frac{i}{n+1}\right]}^{2}.$$

Using Eqs (37), (38), (39) and (40), the partial derivatives of *V*(*β*, *ϑ*, *ζ*) with respect to the parameters *β*, *ϑ* and *ζ* are

$$\frac{\partial V(\beta ,\vartheta ,\zeta )}{\partial \beta }=2\sum_{i=1}^{n}F_{\beta_{i}}\left[F_{i}-\frac{i}{n+1}\right],$$

$$\frac{\partial V(\beta ,\vartheta ,\zeta )}{\partial \vartheta }=2\sum_{i=1}^{n}F_{\vartheta_{i}}\left[F_{i}-\frac{i}{n+1}\right],$$

$$\frac{\partial V(\beta ,\vartheta ,\zeta )}{\partial \zeta }=2\sum_{i=1}^{n}F_{\zeta_{i}}\left[F_{i}-\frac{i}{n+1}\right].$$

When solving the equations $\frac{\partial V(\beta ,\vartheta ,\zeta )}{\partial \beta }=0$, $\frac{\partial V(\beta ,\vartheta ,\zeta )}{\partial \vartheta }=0$ and $\frac{\partial V(\beta ,\vartheta ,\zeta )}{\partial \zeta }=0$, we get the estimates of *β*, *ϑ* and *ζ*.

### 6.5 Weighted least squares method

WLSE extends LSE by introducing weights, giving more importance to certain data points, typically in the tails. It is selected for its flexibility and ability to provide better estimates in scenarios where some observations are more reliable or important than others. The WLSE method [35] depends on minimizing the function

$$W\left(\beta ,\vartheta ,\zeta \right)=\sum_{i=1}^{n}\frac{{\left(n+1\right)}^{2}\left(n+2\right)}{i(n-i+1)}{\left[F\left(y_{\left(i\right)}\right)-\frac{i}{n+1}\right]}^{2}.$$

Again, by using Eqs (37), (38), (39) and (40), the partial derivatives with respect to the parameters *β*, *ϑ* and *ζ* are obtained as:

$$\frac{\partial W(\beta ,\vartheta ,\zeta )}{\partial \beta }=2\sum_{i=1}^{n}\frac{{\left(n+1\right)}^{2}\left(n+2\right)F_{\beta_{i}}}{i(n-i+1)}\left[F_{i}-\frac{i}{n+1}\right],$$

$$\frac{\partial W(\beta ,\vartheta ,\zeta )}{\partial \vartheta }=2\sum_{i=1}^{n}\frac{{\left(n+1\right)}^{2}\left(n+2\right)F_{\vartheta_{i}}}{i(n-i+1)}\left[F_{i}-\frac{i}{n+1}\right],$$

$$\frac{\partial W(\beta ,\vartheta ,\zeta )}{\partial \zeta }=2\sum_{i=1}^{n}\frac{{\left(n+1\right)}^{2}\left(n+2\right)F_{\zeta_{i}}}{i(n-i+1)}\left[F_{i}-\frac{i}{n+1}\right].$$

After solving the system of equations $\frac{\partial W(\beta ,\vartheta ,\zeta )}{\partial \beta }=0$, $\frac{\partial W(\beta ,\vartheta ,\zeta )}{\partial \vartheta }=0$, and $\frac{\partial W(\beta ,\vartheta ,\zeta )}{\partial \zeta }=0$, we get the point estimates of *β*, *ϑ* and *ζ*.

### 6.6 Minimum spacing absolute-log distance

MSALDE minimizes the absolute logarithmic distance between observed and expected spacings, providing robustness against outliers. It is chosen for its effectiveness in dealing with outliers and small sample sizes. The MSALDE aims to minimize the function

$$\Upsilon (\beta ,\vartheta ,\zeta )=\sum_{i=1}^{n+1}\left|\;\text{log}\Lambda_{i}-\;\text{log}\frac{1}{n+1}\right|,$$

where *Λ*_*i*_ = *F*(*y*_(*i*)_) − *F*(*y*_(*i*−1)_). Following the same steps explained above, we can estimate the parameters *β*, *ϑ* and *ζ*.

### 6.7 Percentile estimation

PE is a non-parametric method that relies on the percentiles of the distribution, making it less dependent on the underlying distributional assumptions. The PE approach was originally introduced by [36, 37], applied to the Weibull distribution and later on also used for other distributions. The function

$$\text{PE}\left(\beta ,\vartheta ,\zeta \right)=\sum_{i=1}^{n}{\left[y_{\left(i\right)}-Q\left(\beta ,\vartheta ,\zeta \right)\right]}^{2}$$

is to be minimized to estimate the parameters *β*, *ϑ* and *ζ* by repeating the same steps as discussed above.

### 6.8 Minimum spacing square-log distance

MSSLE minimizes the square of the logarithmic spacing distances, similar to MSALDE, but with a squared term that can offer different sensitivity to deviations. The MSSLE technique aims to minimize the function

$$\delta_{\left(\beta ,\vartheta ,\zeta \right)}=\sum_{i=1}^{n+1}(\;\text{log}(\Lambda_{i}\left(y_{\left(i\right)}\right))-\;\text{log}\frac{1}{n+1}{)}^{2},$$

where *Λ*_*i*_(*y*_*i*_) = *F*(*y*_*i*_) − *F*(*y*_*i*−1_). Following the same steps explained above, we can estimate the parameters *β*, *ϑ* and *ζ*.

## 7 Simulation

In this section, we employ the software R to conduct a Monte Carlo simulation study, aiming to evaluate the performance of the eight estimation methods outlined in Section 6. Specifically, we calculate the point estimate (mean) and determine the mean square error (MSE) as well as the square root of the mean square error (RMSE) for the parameters *β*, *ϑ*, and *ζ*. These metrics provide a comprehensive assessment of the estimators’ accuracy, bias, and efficiency. To achieve this, we generate random samples of different sizes (*n* = 20, 50, 100, 200, 320, 450) from the MKWD. Each sample size was chosen to represent different scenarios, from small to large sample conditions, allowing us to evaluate the estimators’ performance across a broad spectrum.

For each combination of parameter values and sample size, we conducted 1000 replications. This number of replications ensures that our simulation results are reliable and provide a stable estimate of the statistical properties of the parameters. The initial values for the parameters *β*, *ϑ*, and *ζ* are {(0.5, 0.5, 0.5), (0.5, 0.5, 0.9), (0.5, 0.9, 0.5), (0.9, 0.5, 0.5), (1.5, 0.5, 0.5), (1.5, 1.5, 0.5)}. They are used in the simulation to cover various distribution shapes and scales, ensuring that our findings are robust and generalized across different scenarios.

**Fig 2:**
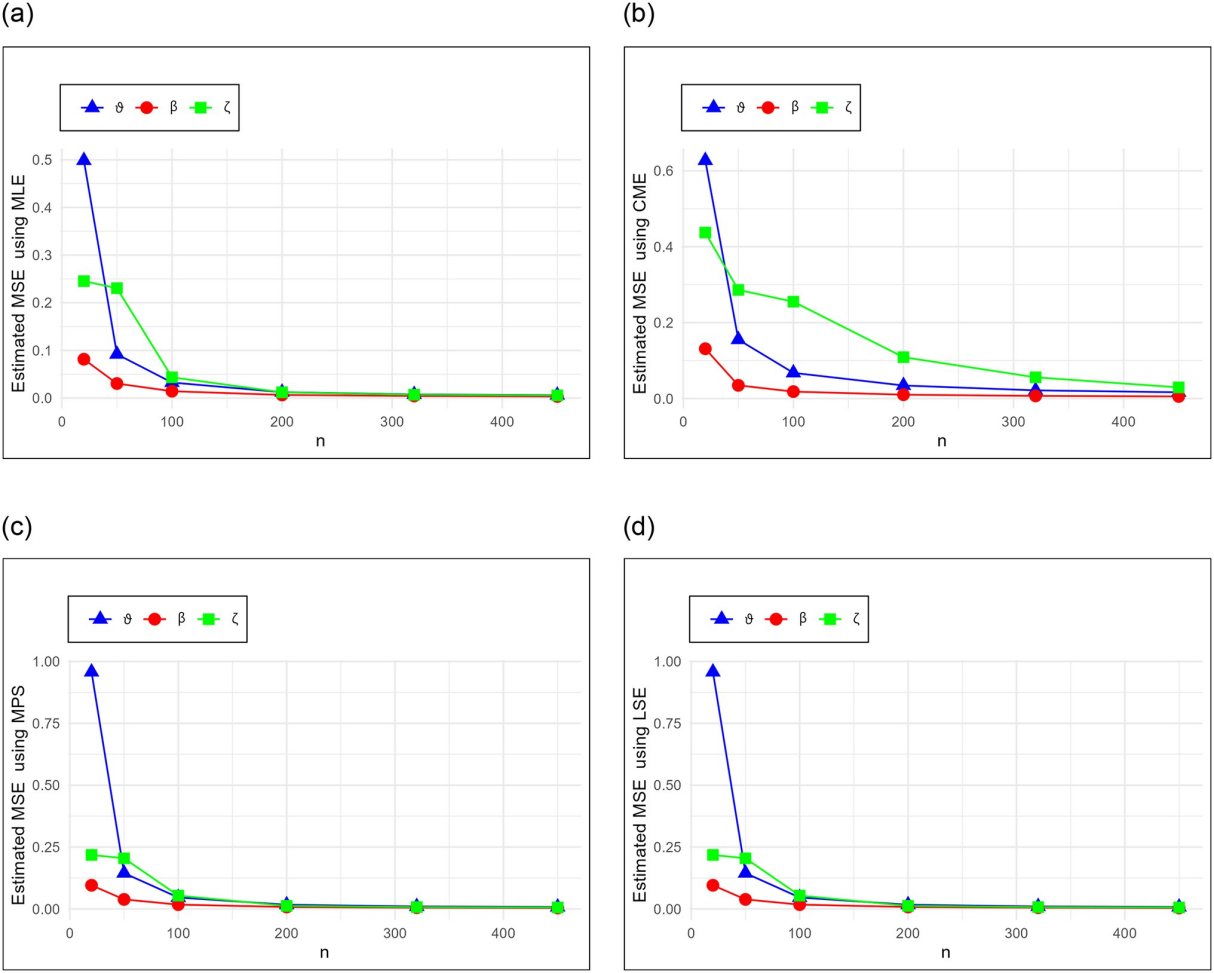
Estimated values of the MSE for MLE, CME, MPSE and LSE schemes in Table 5.

**Fig 3:**
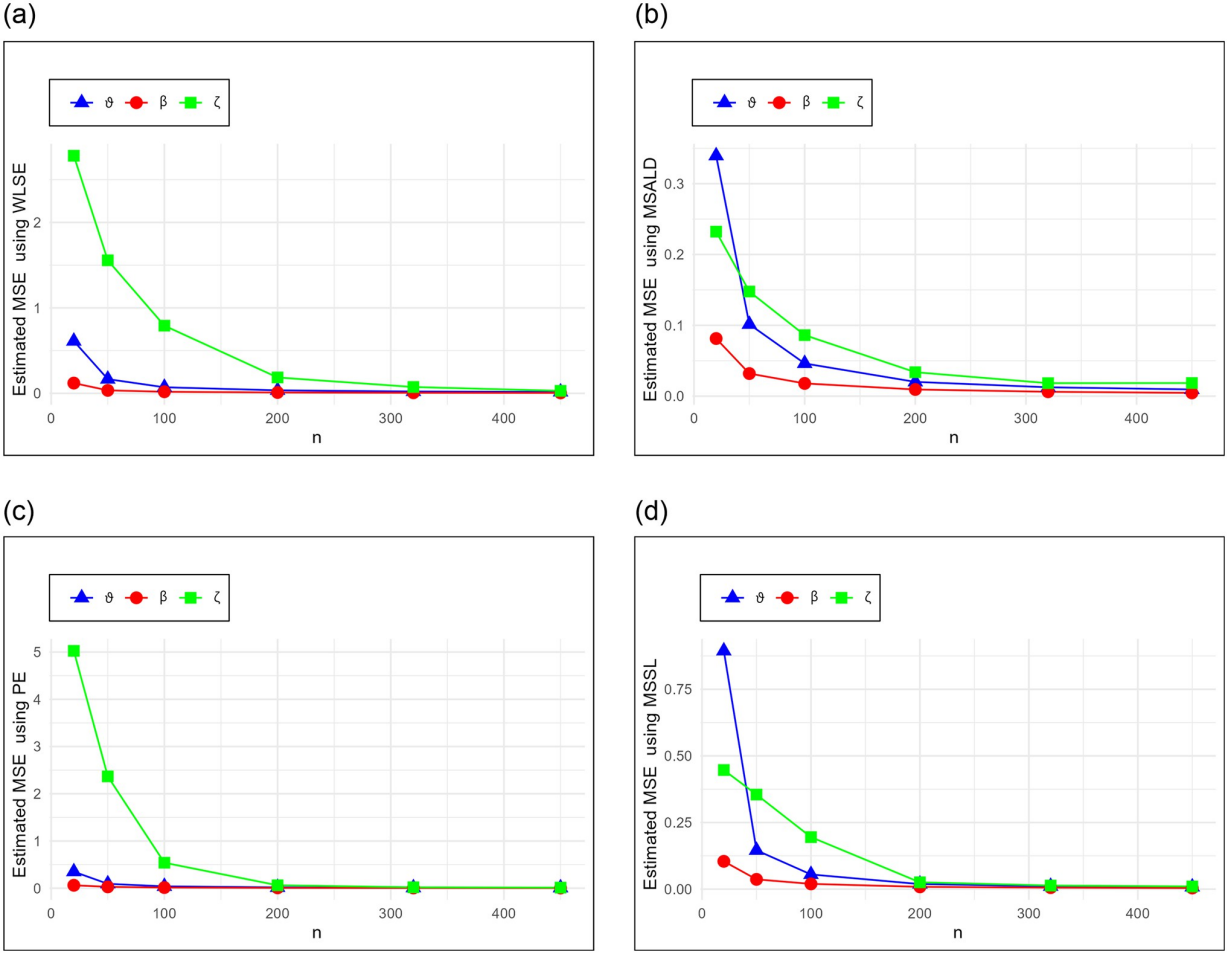
Estimated values of the MSE for WLSE, MSALDE, PE and MSSLE schemes in Table 5.

The results for the mean, MSE, and RMSE for each estimation method and parameter combination are presented in Tables 5–10. These tables allow for a comprehensive comparison of the performance of the different estimation methods. Additionally, the MSE results in Table 5 are graphically displayed in Figs 2 and 3 to illustrate the trend of the estimators as the sample size increases.

**Table 5:** Results for the eight estimation methods considering *ϑ* = 0.5, *β* = 0.5 and *ζ* = 0.5.

*n*		20			50			100			200			320			450		
Method		Mean	MSE	RMSE	Mean	MSE	RMSE	Mean	MSE	RMSE	Mean	MSE	RMSE	Mean	MSE	RMSE	Mean	MSE	RMSE
MLE	*ϑ*	0.9189	0.4986	0.7061	0.6325	0.0920	0.3033	0.5645	0.0326	0.1805	0.5400	0.0123	0.1111	0.5336	0.0076	0.0869	0.5318	0.0061	0.0781
	*β*	0.3748	0.0814	0.2854	0.4463	0.0304	0.1745	0.4726	0.0143	0.1198	0.4797	0.0065	0.0808	0.4817	0.0044	0.0664	0.4838	0.0034	0.0579
	*ζ*	0.4601	0.2454	0.4954	0.5296	0.2307	0.4803	0.4998	0.0435	0.2087	0.4882	0.0121	0.1098	0.4850	0.0070	0.0836	0.4828	0.0056	0.0751
CME	*ϑ*	0.9045	0.6272	0.7920	0.6308	0.1553	0.3941	0.5340	0.0677	0.2602	0.5121	0.0345	0.1856	0.5030	0.0217	0.1472	0.5074	0.0166	0.1287
	*β*	0.4186	0.1313	0.3623	0.4588	0.0349	0.1867	0.4919	0.0183	0.1354	0.4961	0.0102	0.1012	0.4990	0.0071	0.0840	0.4976	0.0056	0.0748
	*ζ*	0.5968	0.4373	0.6613	0.6151	0.2863	0.5350	0.6497	0.2553	0.5052	0.5834	0.1091	0.3303	0.5563	0.0560	0.2366	0.5322	0.0295	0.1717
MPSE	*ϑ*	1.1518	0.9574	0.9785	0.7171	0.1446	0.3803	0.6100	0.0462	0.2149	0.5665	0.0168	0.1295	0.5514	0.0098	0.0989	0.5460	0.0077	0.0876
	*β*	0.3316	0.0955	0.3091	0.4151	0.0384	0.1959	0.4519	0.0173	0.1317	0.4664	0.0078	0.0882	0.4724	0.0051	0.0713	0.4763	0.0038	0.0618
	*ζ*	0.4090	0.2181	0.4671	0.4946	0.2046	0.4523	0.4775	0.0538	0.2319	0.4725	0.0123	0.1110	0.4739	0.0073	0.0856	0.4736	0.0060	0.0773
LSE	*ϑ*	0.8851	0.6470	0.8044	0.6251	0.1555	0.3943	0.5328	0.0679	0.2606	0.5121	0.0345	0.1856	0.5026	0.0218	0.1477	0.5074	0.0166	0.1287
	*β*	0.4288	0.1294	0.3597	0.4611	0.0348	0.1866	0.4924	0.0184	0.1355	0.4961	0.0102	0.1011	0.4991	0.0071	0.0841	0.4976	0.0056	0.0748
	*ζ*	0.6815	0.7145	0.8453	0.6418	0.3709	0.6090	0.6532	0.2612	0.5111	0.5839	0.1110	0.3332	0.5591	0.0639	0.2529	0.5322	0.0295	0.1717
WLSE	*ϑ*	0.8299	0.6114	0.7819	0.5970	0.1668	0.4084	0.5199	0.0714	0.2672	0.5090	0.0355	0.1885	0.5021	0.0220	0.1483	0.5074	0.0166	0.1287
	*β*	0.4409	0.1202	0.3466	0.4728	0.0353	0.1878	0.4976	0.0188	0.1370	0.4974	0.0104	0.1020	0.4993	0.0071	0.0843	0.4976	0.0056	0.0748
	*ζ*	1.0110	2.7806	1.6675	0.8838	1.5572	1.2479	0.7625	0.7921	0.8900	0.6054	0.1861	0.4314	0.5623	0.0745	0.2729	0.5322	0.0295	0.1717
MSALDE	*ϑ*	0.8087	0.3395	0.5826	0.6412	0.1014	0.3185	0.5541	0.0461	0.2148	0.5223	0.0201	0.1419	0.5138	0.0125	0.1117	0.5166	0.0095	0.0972
	*β*	0.4654	0.0814	0.2852	0.4571	0.0319	0.1786	0.4865	0.0179	0.1339	0.4904	0.0094	0.0968	0.4921	0.0062	0.0784	0.4937	0.0046	0.0676
	*ζ*	0.4891	0.2323	0.4819	0.5025	0.1477	0.3843	0.5332	0.0862	0.2936	0.5223	0.0338	0.1838	0.5143	0.0183	0.1353	0.5044	0.0185	0.1360
PE	*ϑ*	0.6900	0.3497	0.5914	0.5258	0.0931	0.3051	0.4996	0.0392	0.1981	0.4970	0.0177	0.1331	0.5001	0.0111	0.1055	0.5073	0.0089	0.0945
	*β*	0.4471	0.0620	0.2490	0.4915	0.0290	0.1702	0.5004	0.0142	0.1191	0.5016	0.0076	0.0870	0.4999	0.0051	0.0717	0.4977	0.0041	0.0642
	*ζ*	1.2825	5.0253	2.2417	0.9910	2.3681	1.5388	0.6739	0.5398	0.7347	0.5580	0.0621	0.2493	0.5290	0.0197	0.1405	0.5137	0.0130	0.1141
MSSLE	*ϑ*	1.0557	0.8941	0.9456	0.6795	0.1457	0.3817	0.5716	0.0550	0.2346	0.5362	0.0189	0.1375	0.5213	0.0107	0.1034	0.5212	0.0083	0.0911
	*β*	0.4062	0.1043	0.3230	0.4504	0.0365	0.1912	0.4771	0.0197	0.1403	0.4845	0.0085	0.0923	0.4910	0.0055	0.0743	0.4921	0.0041	0.0642
	*ζ*	0.4951	0.4469	0.6685	0.5363	0.3548	0.5957	0.5505	0.1952	0.4419	0.5058	0.0255	0.1597	0.5034	0.0135	0.1160	0.4967	0.0101	0.1004

**Table 6:** Results for the eight estimation methods considering *ϑ* = 0.5, *β* = 0.5 and *ζ* = 0.9.

*n*		20			50			100			200			320			450		
Method		Mean	MSE	RMSE	Mean	MSE	RMSE	Mean	MSE	RMSE	Mean	MSE	RMSE	Mean	MSE	RMSE	Mean	MSE	RMSE
MLE	*ϑ*	1.2454	1.2624	1.1236	0.7470	0.2460	0.4960	0.5958	0.0788	0.2807	0.5592	0.0356	0.1886	0.5535	0.0218	0.1476	0.5473	0.0155	0.1246
	*β*	0.3363	0.0941	0.3068	0.4223	0.0295	0.1716	0.4670	0.0131	0.1146	0.4783	0.0067	0.0819	0.4786	0.0042	0.0649	0.4821	0.0031	0.0556
	*ζ*	0.6751	0.7659	0.8752	0.9214	0.7111	0.8433	1.0369	0.7550	0.8689	0.9505	0.3039	0.5513	0.8962	0.1303	0.3610	0.8740	0.0528	0.2297
CME	*ϑ*	1.1962	1.3136	1.1461	0.8034	0.4003	0.6327	0.6690	0.1850	0.4301	0.5627	0.0719	0.2681	0.5302	0.0533	0.2309	0.5152	0.0368	0.1918
	*β*	0.3697	0.0784	0.2800	0.4194	0.0344	0.1854	0.4510	0.0205	0.1432	0.4816	0.0101	0.1003	0.4909	0.0078	0.0882	0.4968	0.0057	0.0758
	*ζ*	0.7942	0.6958	0.8341	0.9728	0.6908	0.8312	1.0070	0.5632	0.7505	1.0571	0.4393	0.6628	1.0709	0.4175	0.6461	1.0308	0.2543	0.5043
MPSE	*ϑ*	1.6409	2.4661	1.5704	0.8846	0.3865	0.6217	0.6718	0.1168	0.3418	0.6015	0.0468	0.2164	0.5819	0.0284	0.1686	0.5690	0.0195	0.1397
	*β*	0.2863	0.1140	0.3376	0.3846	0.0392	0.1981	0.4416	0.0173	0.1317	0.4630	0.0082	0.0906	0.4680	0.0052	0.0721	0.4739	0.0037	0.0605
	*ζ*	0.5706	0.6731	0.8204	0.8231	0.6721	0.8198	0.9625	0.7836	0.8852	0.8954	0.2736	0.5231	0.8666	0.1437	0.3791	0.8483	0.0541	0.2326
LSE	*ϑ*	1.1887	1.4408	1.2003	0.8041	0.4461	0.6679	0.6646	0.1846	0.4297	0.5586	0.0726	0.2694	0.5316	0.0530	0.2302	0.5156	0.0367	0.1915
	*β*	0.3738	0.0757	0.2751	0.4221	0.0351	0.1873	0.4527	0.0205	0.1433	0.4832	0.0102	0.1009	0.4904	0.0077	0.0879	0.4967	0.0057	0.0757
	*ζ*	0.8989	1.1393	1.0674	1.0240	0.8770	0.9365	1.0272	0.6153	0.7844	1.0818	0.5130	0.7163	1.0600	0.3786	0.6153	1.0297	0.2571	0.5070
WLSE	*ϑ*	1.1872	1.5672	1.2519	0.7531	0.4282	0.6544	0.6228	0.1842	0.4292	0.5374	0.0770	0.2775	0.5206	0.0558	0.2362	0.5088	0.0388	0.1969
	*β*	0.3809	0.0791	0.2813	0.4388	0.0347	0.1863	0.4676	0.0210	0.1448	0.4913	0.0109	0.1045	0.4946	0.0082	0.0903	0.4993	0.0061	0.0779
	*ζ*	1.2807	4.2572	2.0633	1.4199	3.2893	1.8136	1.3657	2.4439	1.5633	1.2739	1.4594	1.2081	1.1559	0.8125	0.9014	1.0922	0.5330	0.7301
MSALDE	*ϑ*	1.0065	0.7352	0.8574	0.7556	0.2653	0.5151	0.6354	0.1126	0.3355	0.5541	0.0488	0.2209	0.5365	0.0316	0.1779	0.5226	0.0232	0.1522
	*β*	0.4375	0.0783	0.2797	0.4364	0.0286	0.1692	0.4587	0.0168	0.1295	0.4836	0.0081	0.0898	0.4865	0.0056	0.0747	0.4931	0.0041	0.0640
	*ζ*	0.7092	0.3967	0.6298	0.8841	0.5215	0.7222	0.9330	0.3860	0.6213	0.9900	0.3335	0.5775	0.9576	0.1951	0.4418	0.9506	0.1208	0.3476
PE	*ϑ*	0.8756	0.8254	0.9085	0.5723	0.2009	0.4482	0.5212	0.0992	0.3150	0.5074	0.0448	0.2117	0.5036	0.0296	0.1721	0.5028	0.0216	0.1470
	*β*	0.4388	0.0777	0.2788	0.4839	0.0275	0.1659	0.4955	0.0157	0.1251	0.4979	0.0078	0.0880	0.4986	0.0053	0.0725	0.5002	0.0039	0.0628
	*ζ*	2.8020	22.4899	4.7424	2.4040	13.8550	3.7222	1.7880	6.4869	2.5469	1.2053	1.2067	1.0985	1.0730	0.5175	0.7194	1.0005	0.1831	0.4280
MSSLE	*ϑ*	1.4797	2.2101	1.4866	0.8498	0.4014	0.6336	0.6510	0.1331	0.3649	0.5644	0.0506	0.2250	0.5407	0.0329	0.1814	0.5328	0.0230	0.1516
	*β*	0.3597	0.0946	0.3076	0.4068	0.0369	0.1922	0.4549	0.0187	0.1369	0.4795	0.0086	0.0927	0.4857	0.0058	0.0760	0.4894	0.0041	0.0643
	*ζ*	0.6161	0.6919	0.8318	0.9044	0.9389	0.9690	1.0302	0.9337	0.9663	0.9995	0.4426	0.6653	0.9758	0.2860	0.5348	0.9382	0.1447	0.3804

**Table 7:** Results for the eight estimation methods considering *ϑ* = 0.5, *β* = 0.9 and *ζ* = 0.5.

*n*		20			50			100			200			320			450		
Method		Mean	MSE	RMSE	Mean	MSE	RMSE	Mean	MSE	RMSE	Mean	MSE	RMSE	Mean	MSE	RMSE	Mean	MSE	RMSE
MLE	*ϑ*	0.9210	0.5604	0.7486	0.6354	0.0980	0.3131	0.5641	0.0327	0.1808	0.5426	0.0144	0.1199	0.5306	0.0078	0.0882	0.5309	0.0055	0.0744
	*β*	0.8857	0.3505	0.5921	0.8762	0.0440	0.2097	0.8866	0.0144	0.1198	0.8923	0.0072	0.0846	0.9015	0.0044	0.0661	0.8974	0.0028	0.0533
	*ζ*	0.4727	0.3187	0.5645	0.5040	0.1189	0.3449	0.5024	0.0497	0.2230	0.4906	0.0148	0.1218	0.4875	0.0075	0.0866	0.4838	0.0050	0.0711
CME	*ϑ*	0.8872	0.6115	0.7820	0.6685	0.2008	0.4481	0.5519	0.0719	0.2682	0.5210	0.0338	0.1839	0.4971	0.0246	0.1568	0.5059	0.0157	0.1252
	*β*	1.0044	0.8087	0.8993	0.8962	0.0941	0.3068	0.8825	0.0176	0.1325	0.8892	0.0092	0.0957	0.8922	0.0052	0.0725	0.8933	0.0034	0.0585
	*ζ*	0.5689	0.3915	0.6257	0.5938	0.2712	0.5208	0.6110	0.1998	0.4470	0.5647	0.0804	0.2835	0.5692	0.0633	0.2516	0.5327	0.0259	0.1609
MPSE	*ϑ*	1.2099	1.2517	1.1188	0.7183	0.1517	0.3895	0.6085	0.0440	0.2097	0.5689	0.0191	0.1381	0.5489	0.0101	0.1005	0.5449	0.0070	0.0835
	*β*	0.9817	0.9848	0.9924	0.8836	0.0570	0.2388	0.8897	0.0167	0.1294	0.8935	0.0081	0.0903	0.9024	0.0048	0.0692	0.8980	0.0030	0.0550
	*ζ*	0.4053	0.2749	0.5243	0.4704	0.1393	0.3732	0.4746	0.0355	0.1883	0.4750	0.0149	0.1219	0.4759	0.0078	0.0884	0.4746	0.0054	0.0732
LSE	*ϑ*	0.8672	0.6113	0.7819	0.6601	0.2031	0.4507	0.5482	0.0708	0.2660	0.5205	0.0340	0.1843	0.4967	0.0247	0.1573	0.5059	0.0157	0.1252
	*β*	1.0149	0.8707	0.9331	0.8933	0.0930	0.3049	0.8824	0.0173	0.1315	0.8891	0.0092	0.0958	0.8920	0.0053	0.0726	0.8933	0.0034	0.0585
	*ζ*	0.6828	0.9020	0.9497	0.6404	0.4273	0.6537	0.6227	0.2257	0.4751	0.5677	0.0900	0.3000	0.5716	0.0693	0.2633	0.5327	0.0259	0.1609
WLSE	*ϑ*	0.8351	0.5961	0.7721	0.6331	0.2031	0.4506	0.5301	0.0773	0.2780	0.5182	0.0348	0.1865	0.4963	0.0249	0.1578	0.5059	0.0157	0.1252
	*β*	0.9903	0.6750	0.8216	0.8859	0.0902	0.3004	0.8758	0.0180	0.1343	0.8883	0.0093	0.0963	0.8919	0.0053	0.0728	0.8933	0.0034	0.0585
	*ζ*	0.8824	2.0834	1.4434	0.8355	1.4565	1.2068	0.7795	0.9475	0.9734	0.5873	0.1779	0.4218	0.5742	0.0763	0.2763	0.5327	0.0259	0.1609
MSALDE	*ϑ*	0.9489	0.6063	0.7786	0.6707	0.1544	0.3929	0.5569	0.0453	0.2127	0.5282	0.0222	0.1489	0.5166	0.0130	0.1142	0.5123	0.0083	0.0910
	*β*	1.1008	1.1212	1.0588	0.8943	0.0701	0.2648	0.8914	0.0219	0.1480	0.8918	0.0108	0.1037	0.8994	0.0065	0.0804	0.8966	0.0042	0.0647
	*ζ*	0.4131	0.1381	0.3717	0.4858	0.1037	0.3220	0.5277	0.0775	0.2785	0.5208	0.0370	0.1924	0.5097	0.0176	0.1329	0.5056	0.0104	0.1022
PE	*ϑ*	0.7311	0.3859	0.6212	0.5226	0.0944	0.3072	0.4983	0.0385	0.1962	0.5066	0.0191	0.1381	0.4966	0.0125	0.1120	0.5047	0.0077	0.0879
	*β*	0.8742	0.2903	0.5388	0.8546	0.0352	0.1877	0.8752	0.0130	0.1139	0.8849	0.0069	0.0830	0.8962	0.0042	0.0649	0.8958	0.0027	0.0522
	*ζ*	1.0507	3.5801	1.8921	0.9640	2.2732	1.5077	0.6624	0.4218	0.6494	0.5519	0.0850	0.2916	0.5358	0.0239	0.1547	0.5150	0.0108	0.1037
MSSLE	*ϑ*	1.1000	1.1272	1.0617	0.6766	0.1400	0.3742	0.5693	0.0495	0.2226	0.5367	0.0207	0.1440	0.5225	0.0121	0.1098	0.5211	0.0074	0.0861
	*β*	1.0804	0.8151	0.9028	0.9128	0.0715	0.2675	0.8959	0.0208	0.1442	0.8966	0.0096	0.0981	0.9006	0.0056	0.0749	0.8997	0.0037	0.0610
	*ζ*	0.4488	0.3242	0.5694	0.5291	0.3169	0.5630	0.5373	0.1302	0.3608	0.5105	0.0305	0.1746	0.5048	0.0161	0.1268	0.4958	0.0081	0.0900

**Table 8:** Results for the eight estimation methods considering *ϑ* = 0.9, *β* = 0.5 and *ζ* = 0.5.

*n*		20			50			100			200			320			450		
Method		Mean	MSE	RMSE	Mean	MSE	RMSE	Mean	MSE	RMSE	Mean	MSE	RMSE	Mean	MSE	RMSE	Mean	MSE	RMSE
MLE	*ϑ*	1.6708	1.5943	1.2627	1.1305	0.2994	0.5471	1.0253	0.1156	0.3400	0.9715	0.0441	0.2099	0.9579	0.0265	0.1629	0.9509	0.0179	0.1338
	*β*	0.3763	0.0832	0.2884	0.4459	0.0315	0.1774	0.4704	0.0148	0.1216	0.4831	0.0071	0.0845	0.4846	0.0045	0.0669	0.4854	0.0031	0.0561
	*ζ*	0.4655	0.3525	0.5937	0.5144	0.1458	0.3819	0.4953	0.0444	0.2107	0.4881	0.0138	0.1174	0.4869	0.0079	0.0889	0.4874	0.0055	0.0743
CME	*ϑ*	1.7061	2.2807	1.5102	1.1390	0.5939	0.7707	0.9940	0.2172	0.4660	0.9305	0.1055	0.3248	0.9105	0.0688	0.2623	0.9020	0.0531	0.2304
	*β*	0.4042	0.0841	0.2901	0.4620	0.0362	0.1902	0.4840	0.0178	0.1335	0.4961	0.0102	0.1012	0.4996	0.0069	0.0833	0.5010	0.0053	0.0728
	*ζ*	0.5438	0.3627	0.6022	0.6439	0.3246	0.5697	0.6084	0.2148	0.4634	0.5709	0.1033	0.3214	0.5497	0.0522	0.2285	0.5416	0.0322	0.1794
MPSE	*ϑ*	2.1273	3.2286	1.7968	1.2941	0.5061	0.7114	1.1130	0.1661	0.4075	1.0170	0.0578	0.2403	0.9920	0.0339	0.1841	0.9761	0.0225	0.1501
	*β*	0.3354	0.1081	0.3288	0.4140	0.0397	0.1993	0.4482	0.0180	0.1343	0.4705	0.0082	0.0908	0.4748	0.0051	0.0715	0.4780	0.0036	0.0597
	*ζ*	0.3876	0.2001	0.4473	0.4728	0.0977	0.3125	0.4709	0.0499	0.2233	0.4732	0.0140	0.1184	0.4746	0.0082	0.0904	0.4782	0.0058	0.0760
LSE	*ϑ*	1.7147	2.5206	1.5876	1.1332	0.5939	0.7707	0.9921	0.2180	0.4669	0.9313	0.1050	0.3240	0.9105	0.0688	0.2623	0.9020	0.0531	0.2304
	*β*	0.4102	0.0854	0.2922	0.4632	0.0362	0.1901	0.4844	0.0179	0.1336	0.4960	0.0102	0.1011	0.4996	0.0069	0.0833	0.5010	0.0053	0.0728
	*ζ*	0.6106	0.6309	0.7943	0.6617	0.3844	0.6200	0.6134	0.2255	0.4748	0.5681	0.0953	0.3087	0.5497	0.0522	0.2285	0.5416	0.0322	0.1794
WLSE	*ϑ*	1.6339	2.4721	1.5723	1.0713	0.5980	0.7733	0.9600	0.2329	0.4826	0.9240	0.1096	0.3310	0.9090	0.0697	0.2640	0.9020	0.0531	0.2304
	*β*	0.4245	0.0830	0.2880	0.4758	0.0358	0.1891	0.4917	0.0184	0.1358	0.4977	0.0104	0.1021	0.5000	0.0070	0.0836	0.5010	0.0053	0.0728
	*ζ*	0.8874	2.2587	1.5029	0.9177	1.7184	1.3109	0.7487	0.8196	0.9053	0.6001	0.2287	0.4782	0.5535	0.0603	0.2455	0.5416	0.0322	0.1794
MSALDE	*ϑ*	1.6058	1.5439	1.2425	1.1354	0.3532	0.5943	1.0296	0.1728	0.4157	0.9433	0.0711	0.2666	0.9315	0.0421	0.2051	0.9208	0.0294	0.1714
	*β*	0.4600	0.1109	0.3331	0.4639	0.0344	0.1855	0.4751	0.0187	0.1368	0.4929	0.0100	0.0998	0.4937	0.0062	0.0785	0.4952	0.0045	0.0669
	*ζ*	0.4393	0.2795	0.5287	0.5286	0.2022	0.4497	0.5182	0.0846	0.2908	0.5209	0.0379	0.1947	0.5106	0.0198	0.1406	0.5101	0.0136	0.1167
PE	*ϑ*	1.2946	1.2147	1.1022	0.9297	0.2864	0.5352	0.9215	0.1289	0.3591	0.9087	0.0601	0.2452	0.9074	0.0362	0.1903	0.9002	0.0272	0.1650
	*β*	0.4416	0.0653	0.2556	0.4933	0.0285	0.1687	0.4975	0.0146	0.1206	0.5010	0.0081	0.0898	0.5004	0.0052	0.0718	0.5014	0.0038	0.0620
	*ζ*	1.2070	5.1576	2.2710	0.9623	2.2067	1.4855	0.6546	0.4850	0.6964	0.5513	0.0713	0.2671	0.5239	0.0194	0.1393	0.5225	0.0136	0.1167
MSSLE	*ϑ*	1.9465	2.9398	1.7146	1.2315	0.5664	0.7526	1.0435	0.1751	0.4185	0.9611	0.0687	0.2622	0.9477	0.0387	0.1967	0.9314	0.0253	0.1589
	*β*	0.4176	0.1253	0.3539	0.4461	0.0412	0.2031	0.4749	0.0187	0.1368	0.4900	0.0094	0.0972	0.4902	0.0058	0.0760	0.4934	0.0040	0.0631
	*ζ*	0.4450	0.3787	0.6154	0.5595	0.3186	0.5644	0.5264	0.1247	0.3532	0.5134	0.0417	0.2042	0.4996	0.0160	0.1264	0.5015	0.0100	0.0999

**Table 9:** Results for the eight estimation methods considering *ϑ* = 1.5, *β* = 0.5 and *ζ* = 0.5.

*n*		20			50			100			200			320			450		
Method		Mean	MSE	RMSE	Mean	MSE	RMSE	Mean	MSE	RMSE	Mean	MSE	RMSE	Mean	MSE	RMSE	Mean	MSE	RMSE
MLE	*ϑ*	2.6193	3.8729	1.9680	1.8872	0.8630	0.9290	1.6976	0.3260	0.5710	1.6309	0.1243	0.3526	1.6024	0.0758	0.2753	1.5828	0.0492	0.2218
	*β*	0.3982	0.0713	0.2671	0.4494	0.0300	0.1732	0.4703	0.0154	0.1243	0.4783	0.0075	0.0866	0.4848	0.0046	0.0679	0.4878	0.0031	0.0558
	*ζ*	0.4732	0.2396	0.4895	0.5200	0.1671	0.4088	0.5060	0.0649	0.2547	0.4864	0.0134	0.1157	0.4838	0.0079	0.0891	0.4859	0.0053	0.0727
CME	*ϑ*	2.6831	5.2859	2.2991	1.9350	1.7284	1.3147	1.6678	0.7210	0.8491	1.5577	0.3429	0.5856	1.4948	0.1872	0.4326	1.5134	0.1308	0.3617
	*β*	0.4173	0.0747	0.2734	0.4595	0.0354	0.1882	0.4796	0.0208	0.1442	0.4924	0.0120	0.1096	0.5039	0.0070	0.0836	0.5011	0.0048	0.0694
	*ζ*	0.5691	0.4298	0.6556	0.6226	0.3160	0.5622	0.6204	0.2198	0.4688	0.5771	0.0977	0.3126	0.5597	0.0639	0.2528	0.5326	0.0291	0.1706
MPSE	*ϑ*	3.4615	8.7674	2.9610	2.1424	1.3411	1.1581	1.8215	0.4318	0.6571	1.7128	0.1673	0.4090	1.6574	0.0960	0.3099	1.6237	0.0614	0.2478
	*β*	0.3472	0.0953	0.3086	0.4181	0.0372	0.1928	0.4518	0.0181	0.1347	0.4645	0.0088	0.0936	0.4752	0.0052	0.0722	0.4806	0.0035	0.0589
	*ζ*	0.3992	0.2170	0.4659	0.4772	0.1326	0.3641	0.4849	0.0627	0.2505	0.4703	0.0140	0.1181	0.4722	0.0083	0.0908	0.4769	0.0056	0.0748
LSE	*ϑ*	2.6768	5.8865	2.4262	1.8974	1.6749	1.2942	1.6585	0.7424	0.8616	1.5563	0.3443	0.5868	1.4949	0.1870	0.4324	1.5134	0.1308	0.3617
	*β*	0.4202	0.0748	0.2735	0.4638	0.0351	0.1872	0.4811	0.0210	0.1451	0.4926	0.0120	0.1097	0.5039	0.0070	0.0835	0.5011	0.0048	0.0694
	*ζ*	0.6664	0.8144	0.9025	0.6607	0.4042	0.6357	0.6382	0.2606	0.5105	0.5808	0.1098	0.3314	0.5590	0.0601	0.2451	0.5326	0.0291	0.1706
WLSE	*ϑ*	2.6122	6.1195	2.4738	1.8011	1.6864	1.2986	1.5987	0.7824	0.8845	1.5427	0.3579	0.5982	1.4911	0.1913	0.4373	1.5134	0.1308	0.3617
	*β*	0.4271	0.0744	0.2728	0.4761	0.0349	0.1868	0.4893	0.0215	0.1467	0.4945	0.0122	0.1106	0.5044	0.0071	0.0840	0.5011	0.0048	0.0694
	*ζ*	0.8746	1.9623	1.4008	0.9090	1.7407	1.3193	0.7949	0.9999	1.0000	0.6159	0.2560	0.5059	0.5668	0.0821	0.2865	0.5326	0.0291	0.1706
MSALDE	*ϑ*	2.4269	3.0465	1.7454	1.9089	1.0452	1.0224	1.6968	0.4579	0.6767	1.5813	0.1930	0.4393	1.5491	0.1204	0.3470	1.5320	0.0754	0.2746
	*β*	0.4894	0.1286	0.3586	0.4615	0.0340	0.1844	0.4775	0.0200	0.1415	0.4879	0.0099	0.0994	0.4941	0.0064	0.0798	0.4978	0.0043	0.0655
	*ζ*	0.4536	0.2352	0.4850	0.5225	0.1864	0.4317	0.5158	0.0635	0.2519	0.5221	0.0431	0.2076	0.5129	0.0225	0.1501	0.5072	0.0119	0.1092
PE	*ϑ*	2.0106	2.3064	1.5187	1.5234	0.7387	0.8595	1.4997	0.3961	0.6294	1.5202	0.1745	0.4177	1.5038	0.1025	0.3202	1.5078	0.0677	0.2602
	*β*	0.4575	0.0576	0.2400	0.5015	0.0260	0.1613	0.5007	0.0163	0.1276	0.4976	0.0088	0.0937	0.5029	0.0052	0.0722	0.5019	0.0036	0.0598
	*ζ*	0.9826	2.9170	1.7079	1.0007	2.4602	1.5685	0.7150	0.6414	0.8009	0.5487	0.0587	0.2424	0.5275	0.0232	0.1522	0.5160	0.0117	0.1084
MSSLE	*ϑ*	3.0474	7.0591	2.6569	2.0169	1.4014	1.1838	1.7115	0.4435	0.6660	1.6266	0.1840	0.4290	1.5817	0.1087	0.3297	1.5472	0.0707	0.2659
	*β*	0.4373	0.0997	0.3158	0.4557	0.0390	0.1975	0.4765	0.0186	0.1362	0.4822	0.0094	0.0970	0.4908	0.0061	0.0778	0.4965	0.0041	0.0642
	*ζ*	0.4948	0.5611	0.7491	0.5440	0.2795	0.5287	0.5393	0.1518	0.3897	0.5071	0.0546	0.2336	0.4972	0.0143	0.1195	0.5011	0.0097	0.0987

**Table 10:** Results for the eight estimation methods considering *ϑ* = 1.5, *β* = 1.5 and *ζ* = 0.5.

*n*		20			50			100			200			320			450		
Method		Mean	MSE	RMSE	Mean	MSE	RMSE	Mean	MSE	RMSE	Mean	MSE	RMSE	Mean	MSE	RMSE	Mean	MSE	RMSE
MLE	*ϑ*	2.8193	4.9833	2.2323	1.9470	1.0136	1.0068	1.6819	0.2795	0.5286	1.6335	0.1174	0.3426	1.6134	0.0708	0.2661	1.5907	0.0484	0.2201
	*β*	2.2739	4.3770	2.0921	1.6808	0.4119	0.6418	1.5917	0.1038	0.3222	1.5711	0.0417	0.2042	1.5560	0.0232	0.1523	1.5453	0.0162	0.1273
	*ζ*	0.4632	0.2977	0.5456	0.5115	0.1549	0.3936	0.5060	0.0401	0.2002	0.4830	0.0123	0.1109	0.4805	0.0069	0.0832	0.4842	0.0052	0.0719
CME	*ϑ*	2.7405	6.3472	2.5194	2.0294	1.7685	1.3298	1.6583	0.7017	0.8377	1.5153	0.2840	0.5329	1.5336	0.2015	0.4489	1.5147	0.1399	0.3740
	*β*	2.6786	11.1416	3.3379	1.8969	1.8830	1.3722	1.6410	0.4449	0.6670	1.5220	0.1182	0.3438	1.5225	0.0794	0.2818	1.5092	0.0499	0.2234
	*ζ*	0.6140	0.5114	0.7151	0.6026	0.3066	0.5538	0.6262	0.2187	0.4677	0.5804	0.0870	0.2949	0.5484	0.0531	0.2304	0.5335	0.0269	0.1641
MPSE	*ϑ*	3.8388	15.6388	3.9546	2.2190	1.6712	1.2928	1.8266	0.4109	0.6410	1.7119	0.1586	0.3983	1.6686	0.0934	0.3057	1.6316	0.0613	0.2476
	*β*	3.0183	11.8048	3.4358	1.8347	0.5535	0.7439	1.6773	0.1541	0.3926	1.6158	0.0563	0.2372	1.5869	0.0303	0.1741	1.5681	0.0204	0.1429
	*ζ*	0.4005	0.2445	0.4945	0.4753	0.1544	0.3930	0.4804	0.0404	0.2009	0.4677	0.0126	0.1122	0.4692	0.0074	0.0860	0.4754	0.0055	0.0740
LSE	*ϑ*	2.7205	6.7632	2.6006	2.0089	1.7394	1.3189	1.6622	0.7003	0.8369	1.5115	0.2879	0.5365	1.5278	0.2070	0.4550	1.5147	0.1399	0.3740
	*β*	2.5918	10.0908	3.1766	1.8783	1.7937	1.3393	1.6433	0.4454	0.6673	1.5199	0.1192	0.3452	1.5192	0.0810	0.2847	1.5092	0.0499	0.2234
	*ζ*	0.6943	0.8328	0.9126	0.6200	0.3429	0.5855	0.6225	0.2133	0.4619	0.5868	0.1015	0.3185	0.5562	0.0674	0.2595	0.5335	0.0269	0.1641
WLSE	*ϑ*	2.5957	6.6199	2.5729	1.8863	1.7638	1.3281	1.5814	0.7547	0.8687	1.5021	0.2983	0.5462	1.5265	0.2085	0.4566	1.5147	0.1399	0.3740
	*β*	2.5008	9.7445	3.1216	1.8095	1.7777	1.3333	1.5960	0.4485	0.6697	1.5147	0.1220	0.3493	1.5184	0.0815	0.2854	1.5092	0.0499	0.2234
	*ζ*	0.9522	2.2684	1.5061	0.9066	1.7834	1.3354	0.8209	1.1292	1.0627	0.6146	0.2263	0.4757	0.5595	0.0784	0.2800	0.5335	0.0269	0.1641
MSALDE	*ϑ*	2.8897	6.3825	2.5264	2.0337	1.3714	1.1711	1.7038	0.4756	0.6897	1.5839	0.1760	0.4195	1.5666	0.1164	0.3411	1.5312	0.0763	0.2762
	*β*	2.7280	10.4285	3.2293	1.7973	0.7992	0.8940	1.6153	0.1934	0.4398	1.5521	0.0728	0.2699	1.5306	0.0408	0.2020	1.5136	0.0274	0.1654
	*ζ*	0.4390	0.2145	0.4631	0.5129	0.2024	0.4499	0.5295	0.0848	0.2913	0.5138	0.0343	0.1852	0.5045	0.0203	0.1423	0.5083	0.0113	0.1065
PE	*ϑ*	2.0782	2.8102	1.6764	1.6236	0.8374	0.9151	1.4969	0.3518	0.5931	1.5011	0.1537	0.3920	1.5236	0.1036	0.3219	1.5134	0.0707	0.2658
	*β*	1.9333	3.6859	1.9199	1.5333	0.3546	0.5955	1.4986	0.1361	0.3689	1.5032	0.0552	0.2350	1.5097	0.0353	0.1879	1.5055	0.0232	0.1523
	*ζ*	1.0803	3.6556	1.9119	0.9088	1.9858	1.4092	0.6881	0.5195	0.7208	0.5512	0.0593	0.2436	0.5217	0.0204	0.1427	0.5155	0.0115	0.1072
MSSLE	*ϑ*	3.4980	12.2682	3.5026	2.1047	1.8517	1.3608	1.7099	0.4436	0.6660	1.6054	0.1716	0.4143	1.5846	0.1003	0.3168	1.5620	0.0661	0.2570
	*β*	3.1844	14.2424	3.7739	1.8334	0.8008	0.8949	1.6426	0.1944	0.4410	1.5680	0.0694	0.2634	1.5476	0.0383	0.1957	1.5343	0.0250	0.1582
	*ζ*	0.4692	0.5099	0.7141	0.5496	0.3262	0.5712	0.5418	0.1408	0.3752	0.5086	0.0347	0.1862	0.4968	0.0143	0.1197	0.4966	0.0086	0.0927

To provide a holistic view, Table 11 summarizes the sum of ranks and overall ranks of the MSE and RMSE across all tables. This comparative analysis highlights the effectiveness of each estimation method. We can deduce the following key findings:

- MSE and RMSE show a decreasing trend with increasing *n* for all estimation methods. This emphasizes the consistency.
- For all parameters (*ϑ*, *β*, *ζ*), the mean estimates converge towards the initial parameter values as *n* increases.
- The overall ranks in Table 11 show that MLE leads to the best results when estimating the parameters.
- MLE is the top-performing method, showing the lowest MSE and RMSE values across all parameters and sample sizes.
- MPSE follows closely behind MLE, with competitive MSE and RMSE values. It performs well across different parameter values, especially with larger sample sizes.
- PE is preferred after MLE and MPSE, however, it may require larger sample sizes to achieve optimal performance.
- The other methods according to their ranks (after MLE, MPSE and PE) are MSALDE (which performs well at small sample sizes), MSSLE, CME, LSE and WLSE.

**Table 11:** Sum of ranks for the results of Tables 5–10.

Parameters	*n*	MLE	CME	MPSE	LSE	WLSE	MSALDE	PE	MSSLE
*ϑ* = 0.5, *β* = 0.5 and *ζ* = 0.5	20	2	6	4	8	6	1	3	6
	50	1	5	4	6.5	8	2	3	6.5
	100	1	5	2	6	8	4	3	7
	200	1	6	2	7	8	5	3	4
	320	1	6	2	7	8	5	4	3
	450	1	7	2	7	7	5	4	3
*ϑ* = 0.5, *β* = 0.5 and *ζ* = 0.9	20	5	3	7.5	3	7.5	1	3	6
	50	2	4	5	7	7	1	3	7
	100	1	5.5	4	7	8	2	3	5.5
	200	1	6	2	7	8	3	4	5
	320	1	7	2	6	8	3	4	5
	450	1	7	2	6	8	4	3	5
*ϑ* = 0.5, *β* = 0.9 and *ζ* = 0.5	20	1	5	7.5	7.5	3.5	3.5	2	6
	50	1	6	3	8	7	3	3	5
	100	1	6	2	5	8	4	3	7
	200	1	5	2	7	8	6	3	4
	320	1	5	2	7	8	6	3	4
	450	1	7	2	7	7	5	3	4
*ϑ* = 0.9, *β* = 0.5 and *ζ* = 0.5	20	1	4	6	7	5	3	2	8
	50	1	7	4	5.5	8	2	3	5.5
	100	1	4	3	6	8	5	2	7
	200	1	7	2	6	8	5	3	4
	320	1	6.5	2	6.5	8	5	3	4
	450	1	7	2	7	7	5	4	3
*ϑ* = 1.5, *β* = 0.5 and *ζ* = 0.5	20	1	3.5	5	6.5	6.5	3.5	2	8
	50	1	8	4	5.5	7	2	3	5.5
	100	1	6	2	7	8	4	3	5
	200	1	6	2	7	8	5	3	4
	320	1	7	2	6	8	5	3	4
	450	1	7	2	7	7	5	3	4
*ϑ* = 1.5, *β* = 1.5 and *ζ* = 0.5	20	1	4	7	6	5	2.5	2.5	8
	50	1	7.5	2	5.5	7.5	3.5	3.5	5.5
	100	1	7	2	6	8	4	3	5
	200	1	6	2	7	8	5	3	4
	320	1	6	2	7	8	5	4	3
	450	1	7	2	7	7	5	4	3
∑ Ranks		42	212	110	234.5	265	138	111	183.5
Overall Ranks		1	6	2	7	8	4	3	5

## 8 Data analysis

**Fig 4:**
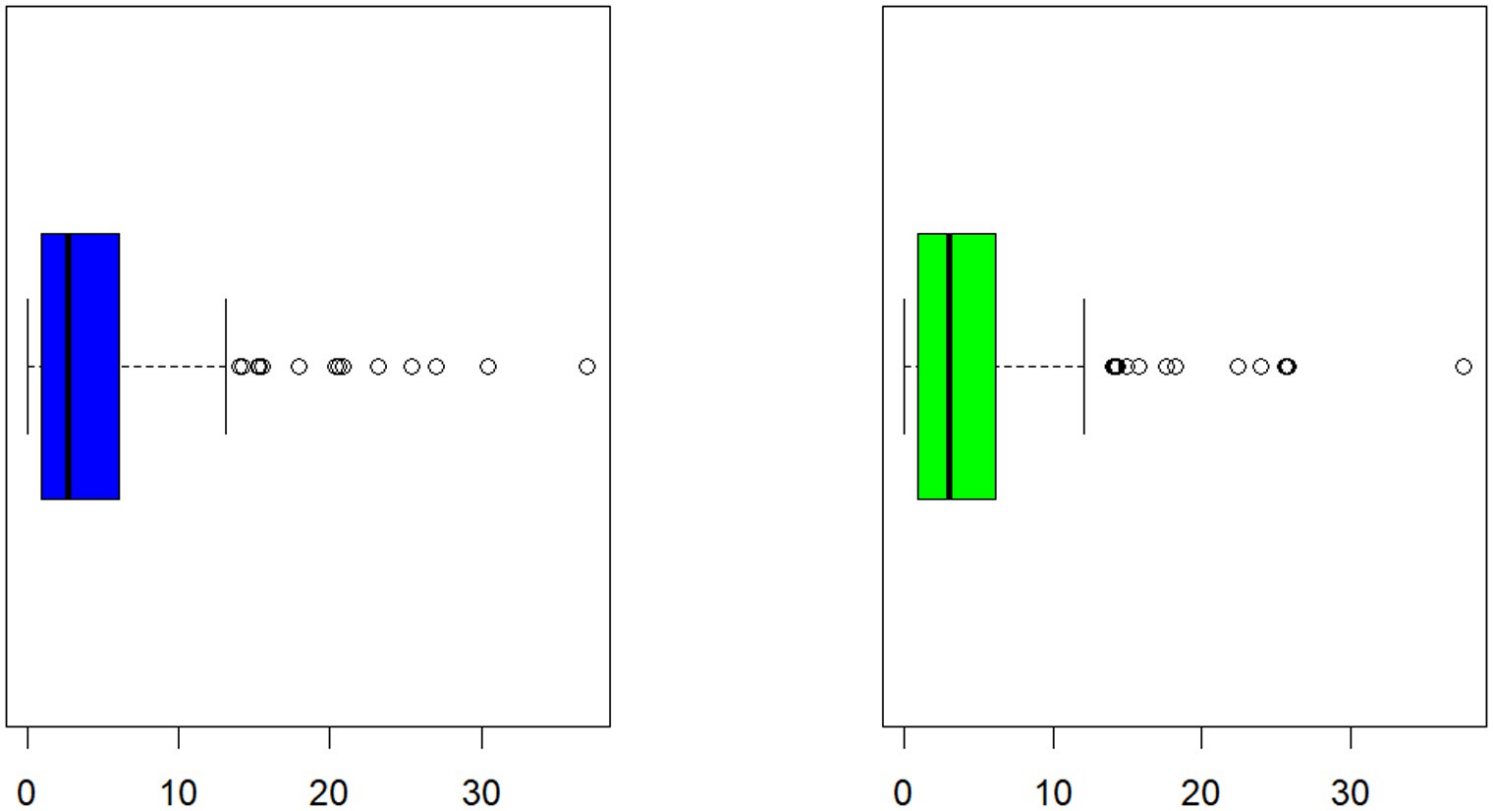
Box plots for both data sets.

**Fig 5:**
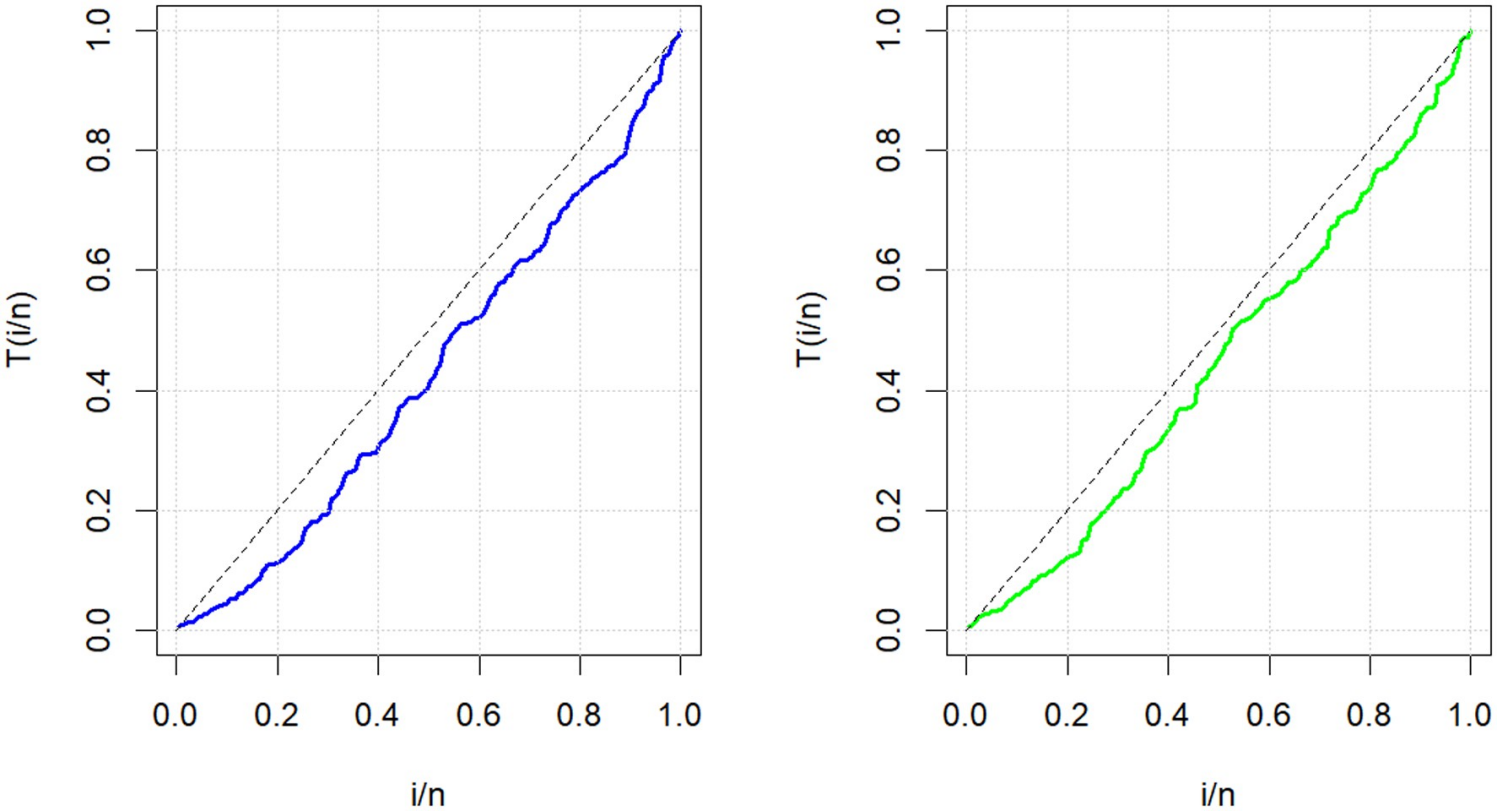
TTT plots for both data sets.

**Fig 6:**
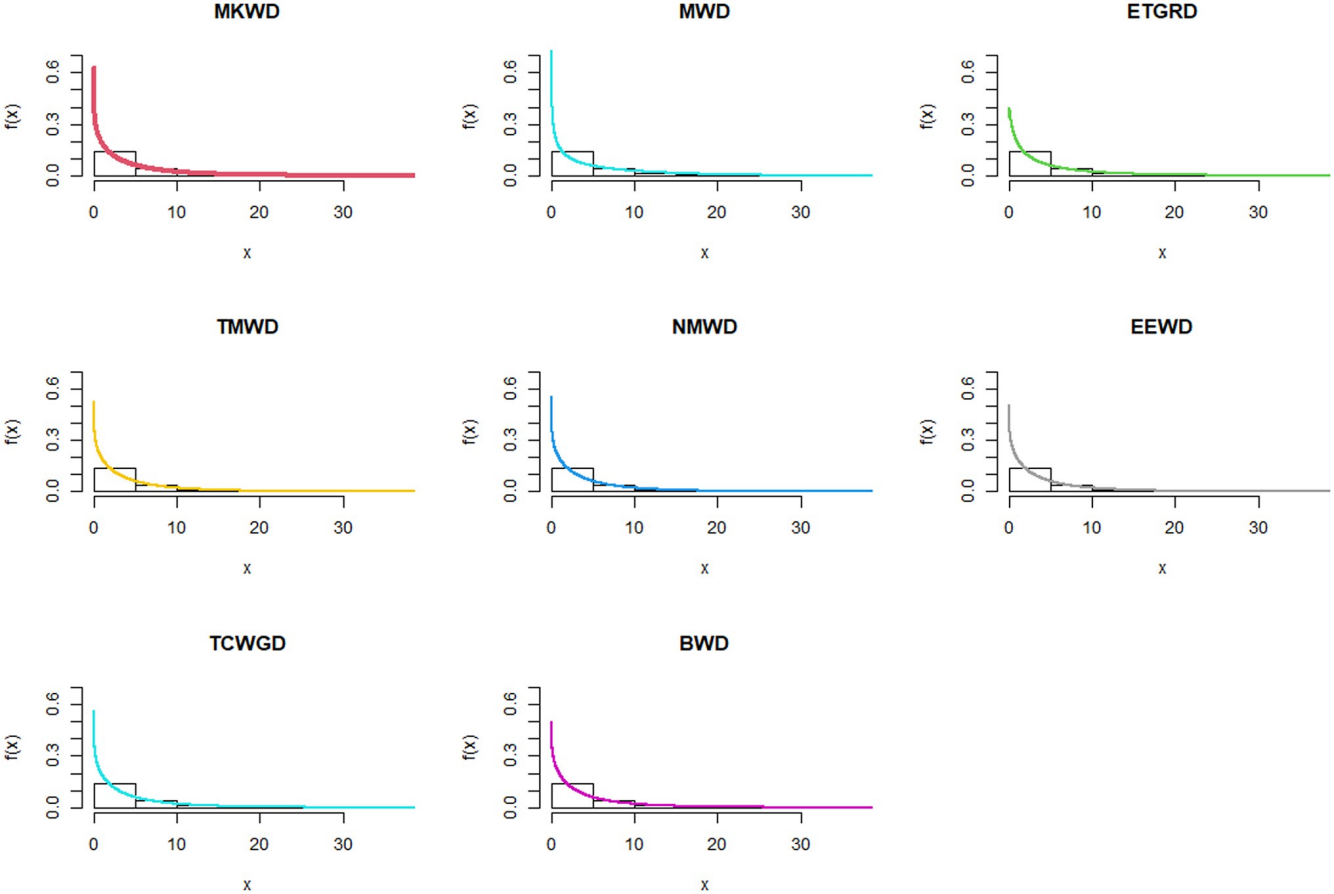
Estimated pdf plots of data set 1.

**Fig 7:**
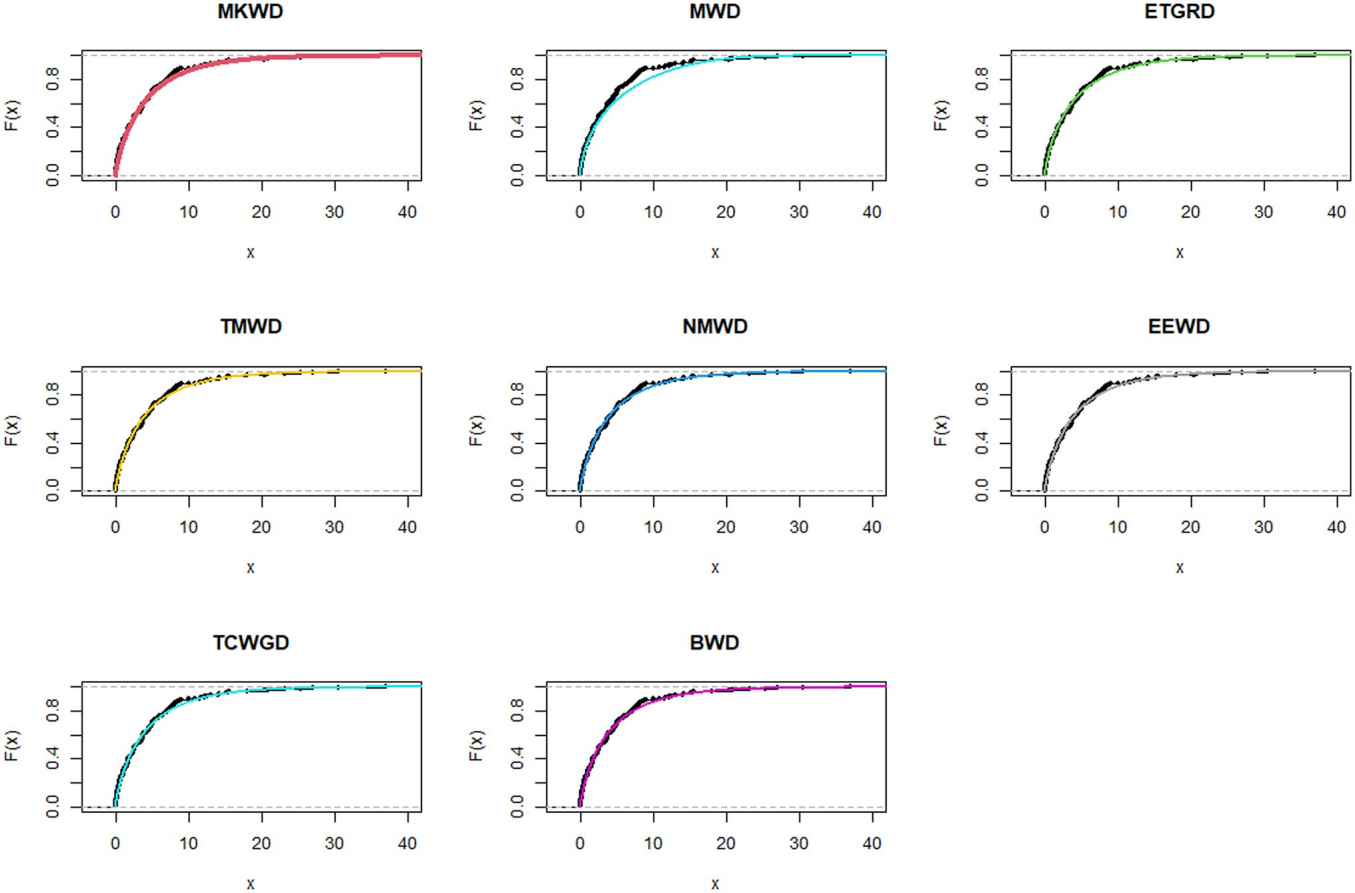
Estimated cdf plots of data set 1.

**Fig 8:**
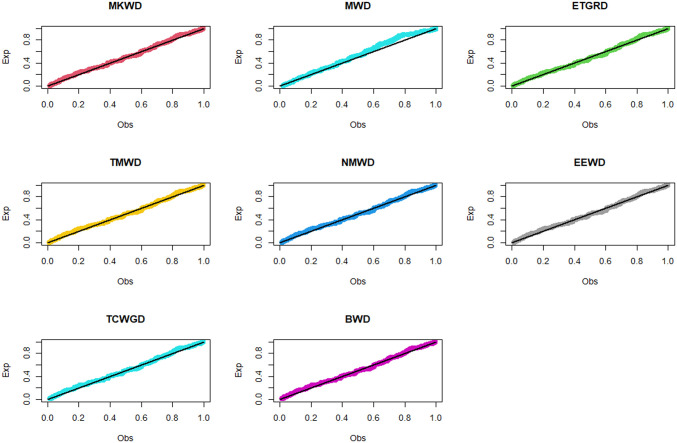
Estimated PP plots of data set 1.

**Fig 9:**
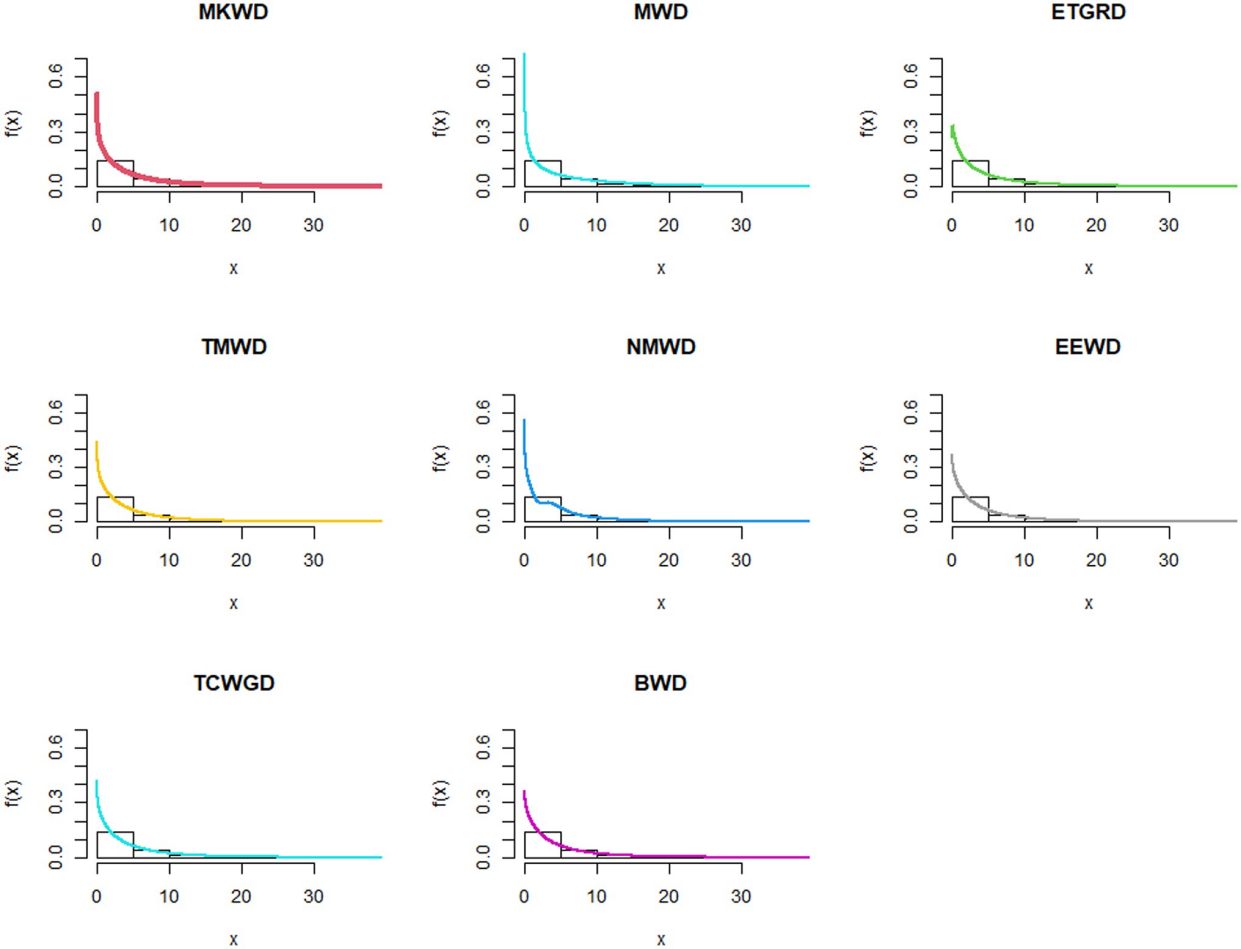
Estimated pdf plots of data set 2.

**Fig 10:**
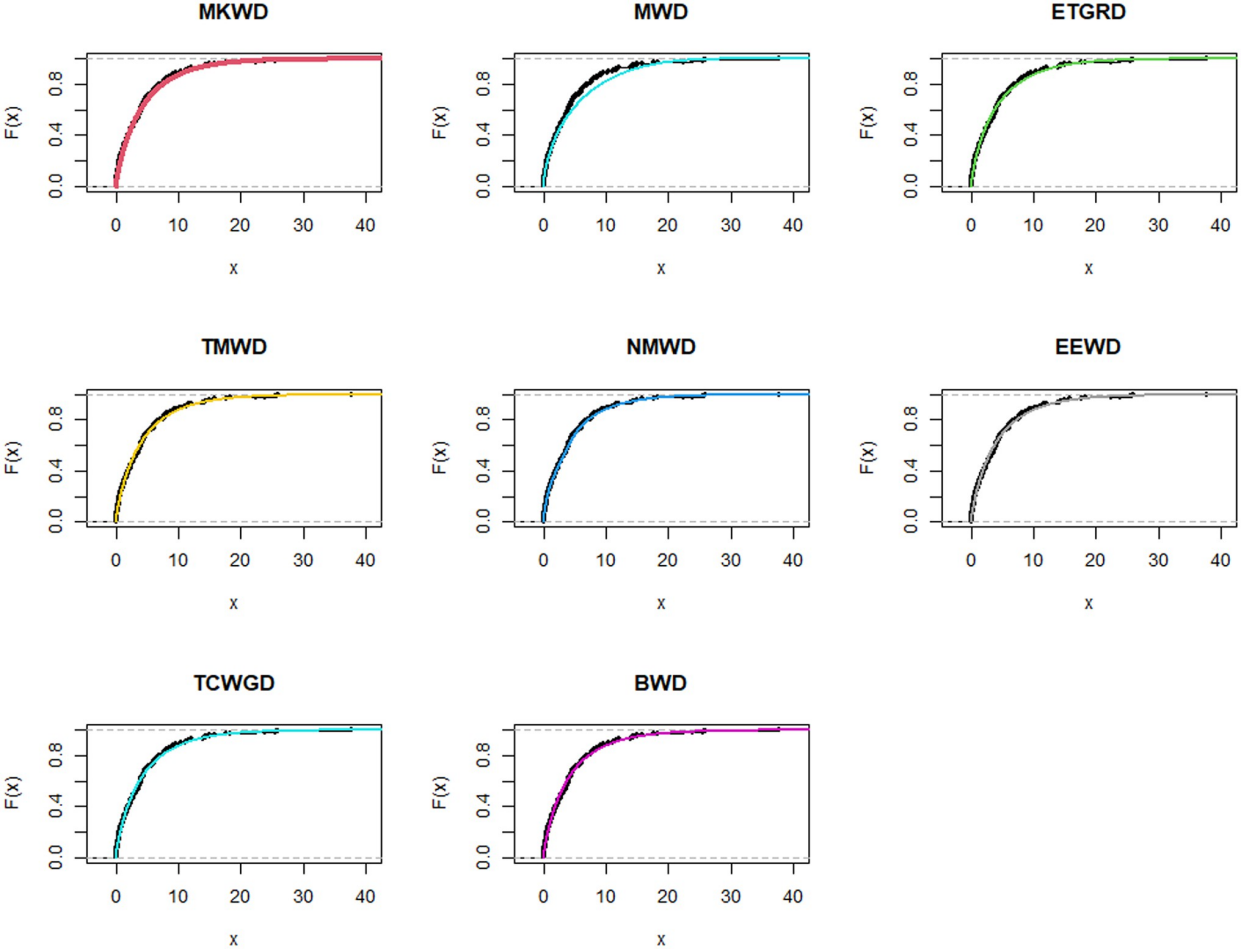
Estimated cdf plots of data set 2.

**Fig 11:**
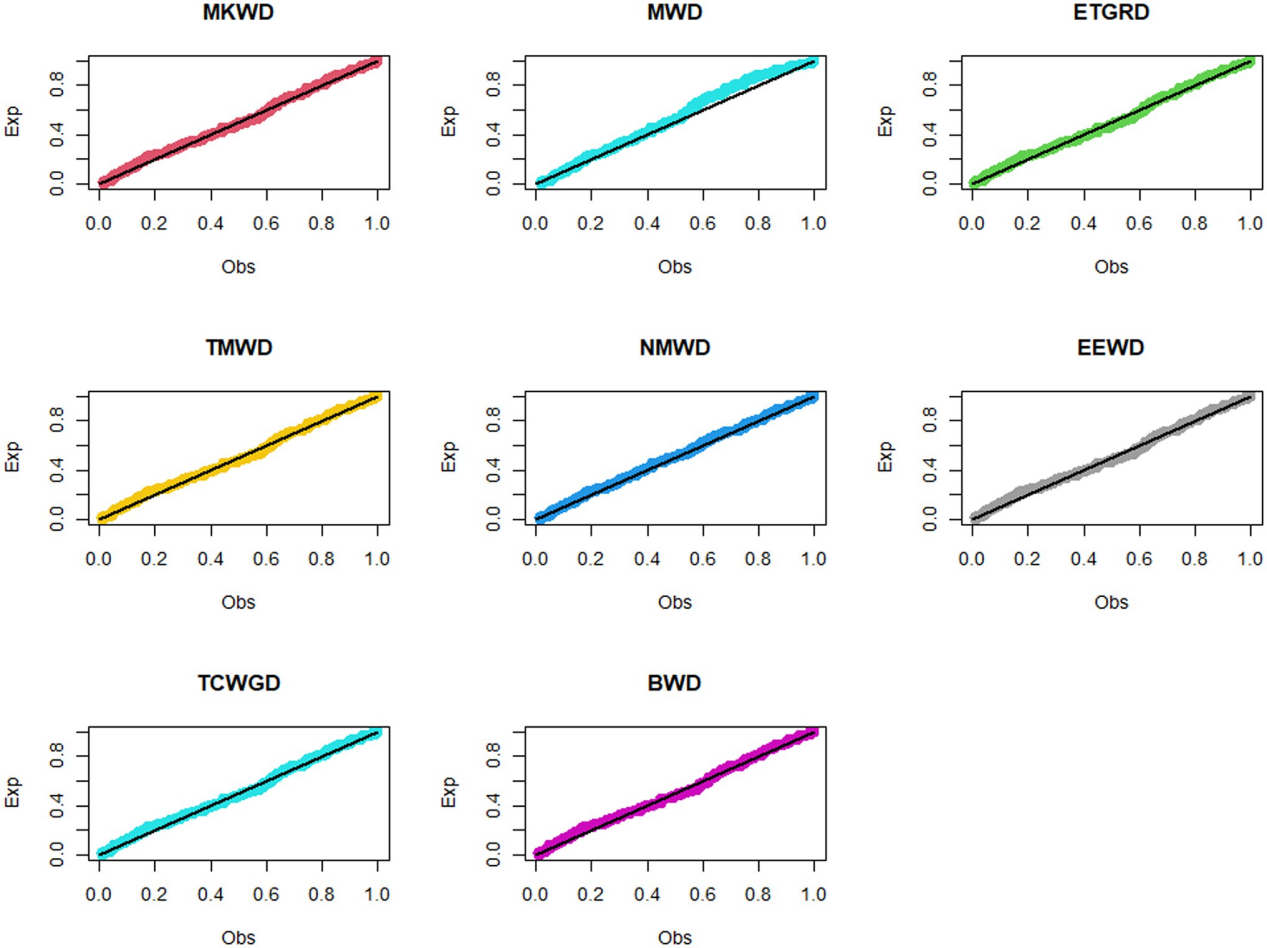
Estimated PP plots of data set 2.

The relevance and importance of the MKWD are demonstrated in this section through the use of two real data sets. The data sets are given in Sections 8.1 and 8.2, and their box plots and the total time on test (TTT) plots can be found in Figs 4 and 5. From Fig 4 we note that both data sets are skewed to right, and from Fig 5 we note that the estimated hrf for both data sets are decreasing. The goodness-of-fit criteria, MLEs of parameters and standard errors (SEs) (we focus on ML as it has turned out to be the best estimation method, see Section 7) of the proposed model are compared to those of other competing models. The competing models are modified WD (MWD) [38], exponentiated transmuted generalized Rayleigh distribution (ETGRD) [39], transmuted modified WD (TMWD) [40], new modified WD (NMWD) [41], exponentiated exponential WD (EEWD) [42], transmuted complementary Weibull geometric distribution (TCWGD) [43] and beta WD (BWD) [44]. Based on the results in Tables 12–15, it is clear that the MKWD exhibits the best modeling ability among the models investigated. This is demonstrated by the lowest Akaike information criterion (*ζ*_1_), Bayesian information criterion (*ζ*_2_), consistency of *ζ*_1_ (*ζ*_3_), Hannan-Quinn information criterion (*ζ*_4_), Kolmogorov-Smirnov test statistic (*ζ*_5_), Cramér-von-Mises test statistic (*ζ*_7_), Anderson-Darling test statistic (*ζ*_8_) values, and largest *p*-value related to *ζ*_5_ (*ζ*_6_). Figs 6–11 (estimated pdf, cdf, PP plots for both data sets) also justify this claim, demonstrating the superiority of the MKWD over its competitors.

**Table 12:** MLEs and SEs for data set 1.

Model	$\hat{\beta }$	$\text{SE}(\hat{\beta })$	$\hat{\vartheta }$	$\text{SE}(\hat{\vartheta })$	$\hat{\zeta }$	$\text{SE}(\hat{\zeta })$	$\hat{\alpha }$	$\text{SE}(\hat{\alpha })$	$\hat{\lambda }$	$SE(\hat{\lambda })$
MKWD	0.6876	0.8146	0.0057	0.0173	106.1439	2.8741				
MWD	0.6570	0.1335	1.1441	0.2889	0.0917	0.0200				
ETGRD	0.0467	0.0189	0.0495	0.0124	18.2450	12.2752	0.9556	0.0540		
TMWD	0.5035	2.6679	0.9402	0.2225	0.8231	2.6489	0.0516	0.4631		
NMWD	0.2552	0.1721	0.0513	0.1701	0.8580	0.1309	0.0385	0.1317	0.8553	0.4374
EEWD	1.1850	0.4015	0.2385	1.3534	0.7567	0.1470	0.1329	0.5768		
TCWGD	1.1862	1.3029	0.8612	0.1202	0.2142	0.0968	0.0009	0.8886		
BWD	0.1240	0.2969	0.7404	0.1750	1.2195	0.4791	1.9909	3.7550		

**Table 13:** Goodness-of-fit criteria for data set 1.

Distribution	*ζ*_1_	*ζ*_2_	*ζ*_3_	*ζ*_4_	*ζ*_5_	*ζ*_6_	*ζ*_7_	*ζ*_8_
MKWD	1057.4012	1067.4568	1057.5171	1061.4658	0.0387	0.9108	0.0546	0.3905
MWD	1071.4420	1081.4980	1071.5580	1075.5070	0.0997	0.0302	0.0991	0.8156
ETGRD	1058.0590	1071.4660	1058.2530	1063.4780	0.0459	0.7660	0.0762	0.4689
TMWD	1059.0120	1072.4190	1059.2060	1064.4310	0.0436	0.8179	0.0655	0.4240
NMWD	1061.1850	1077.9450	1061.4780	1067.9600	0.0428	0.8333	0.0646	0.4237
EEWD	1059.0710	1072.4780	1059.2650	1064.4900	0.0448	0.7919	0.0696	0.4419
TCWGD	1059.2670	1072.6750	1059.4620	1064.6870	0.0419	0.8533	0.0629	0.4197
BWD	1059.0530	1072.4610	1059.2470	1064.4730	0.0450	0.7858	0.0706	0.4456

**Table 14:** MLEs and SEs for data set 2.

Model	$\hat{\beta }$	$\text{SE}(\hat{\beta })$	$\hat{\vartheta }$	$\text{SE}(\hat{\vartheta })$	$\hat{\zeta }$	$\text{SE}(\hat{\zeta })$	$\hat{\alpha }$	$\text{SE}(\hat{\alpha })$	$\hat{\lambda }$	$\text{SE}(\hat{\lambda })$
MKWD	0.6842	0.1048	0.0088	0.0001	70.7497	0.8073				
MWD	0.6603	0.1305	1.1961	0.2951	0.0952	0.0202				
ETGRD	0.0662	0.0364	0.0538	0.0110	11.3056	10.0106	0.9484	0.0558		
TMWD	1.3674	4.2841	0.9771	0.0616	1.6696	4.3035	0.0662	0.5141		
NMWD	0.2180	0.1875	0.0655	0.1838	0.9030	0.1501	0.0338	0.1230	0.8984	0.3600
EEWD	1.2412	0.4250	1.8171	22.5763	0.7708	0.1495	0.6205	5.9609		
TCWGD	1.2674	1.3569	0.9113	0.1243	0.2006	0.0850	0.0001	0.8527		
BWD	0.1008	0.3704	0.7474	0.1840	1.2918	0.5278	2.4378	7.1105		

**Table 15:** Goodness-of-fit criteria for data set 2.

Distribution	*ζ*_1_	*ζ*_2_	*ζ*_3_	*ζ*_4_	*ζ*_5_	*ζ*_6_	*ζ*_7_	*ζ*_8_
MKWD	1077.9050	1088.0030	1078.0200	1081.9860	0.0436	0.8105	0.0543	0.3893
MWD	1092.9150	1103.0130	1093.0290	1096.9950	0.0858	0.0854	0.0968	0.7903
ETGRD	1078.6220	1092.0860	1078.8130	1084.0620	0.0488	0.6890	0.0664	0.4148
TMWD	1079.3850	1092.8490	1079.5760	1084.8250	0.0496	0.6684	0.0645	0.4121
NMWD	1081.5420	1098.3720	1081.8310	1088.3430	0.0497	0.6657	0.0685	0.4312
EEWD	1079.3610	1092.8250	1079.5520	1084.8010	0.0525	0.5973	0.0756	0.4560
TCWGD	1079.6540	1093.1180	1079.8450	1085.0940	0.0493	0.6753	0.0678	0.4301
BWD	1079.3290	1092.7930	1079.5200	1084.7690	0.0528	0.5889	0.0766	0.4593

### 8.1 Data set 1: proportion of global per capita CO_2_ emissions in 2020

The first data set shows the proportion of global CO_2_ emissions per person for 211 nations in 2020. The data set, which was previously utilized in [45], is given by: 0.18, 1.88, 0.58, 3.53, 20.32, 5.39, 7.41, 0.11, 0.68, 2.09, 0.71, 0.26, 0.26, 0.21, 3.8, 0.73, 3.78, 0.99, 0.31, 2.16, 1.76, 5.01, 11.47, 6.53, 0.94, 3.37, 1.93, 6.08, 7.69, 0.67, 5, 0.04, 15.37, 0.56, 4.85, 14, 6.75, 4.66, 9.06, 1.68, 2.62, 2.56, 0.36, 15.52, 1.36, 0.57, 1.75, 0.08, 6.04, 1.75, 3.32, 8.6, 2.5, 2.56, 6.26, 0.92, 0.03, 7.62, 17.97, 0.59, 1.99, 1.53, 1.06, 0.4, 5.63, 5.24, 8.42, 6.94, 0.43, 4.89, 7.09, 3.47, 13.06, 0.64, 8.15, 1.02, 0.13, 3.99, 12.12, 0.43, 5.07, 2.5, 1.14, 0.04, 5.94, 1.06, 4.47, 0.07, 4.99, 1.93, 8.23, 0.38, 1.24, 5.02, 1.47, 6.73, 0.51, 30.45, 0.36, 20.55, 12.17, 0.77, 0.62, 26.98, 2.36, 3.96, 2.38, 4.24, 2.4, 1.56, 3.79, 2.44, 2.98, 7.32, 0.07, 4.65, 3.43, 6.51, 0.2, 3.61, 23.22, 12.49, 0.99, 15.19, 3.83, 0.26, 7.05, 2.77, 14.24, 4.25, 4.94, 2.51, 0.05, 0.98, 0.15, 3.72, 1.55, 7.62, 2.5, 5.07, 0.06, 0.3, 1.24, 6.98, 5.23, 1.55, 10.81, 2.2, 1.77, 0.11, 7.92, 6.4, 2.81, 11.66, 6.03, 2.95, 1.74, 0.56, 1.36, 0.61, 0.74, 0.17, 3.7, 0.99, 0.11, 8.87, 0.21, 2.77, 0.2, 4.52, 25.37, 14.2, 5.24, 20.83, 1.28, 3.69, 0.82, 3.59, 1.78, 8.06, 5.38, 3.73, 8.22, 7.23, 2.5, 3.68, 1.77, 0.33, 0.13, 0.55, 4.52, 0.19, 1.06, 2.61, 4.14, 1.58, 37.02, 8.74, 4.4, 4.61, 7.88, 0.51, 1.75, 10.03, 3.72, 1.94, 0.3, 3.13, 0.26, 7.78, 7.38.

### 8.2 Data set 2: proportion of global per capita CO_2_ emissions in 2022

The second data set comprises the proportion of global CO_2_ emissions per person for 214 nations in 2022. The electronic address from where it is taken is as follows: https://ourworldindata.org/. The data set consists of the following values: 0.295, 1.743, 3.927, 4.617, 0.452, 8.753, 6.422, 4.238, 2.305, 8.133, 14.985, 6.878, 3.675, 5.171, 25.672, 0.596, 4.377, 6.167, 7.688, 1.789, 0.631, 6.937, 1.349, 1.758, 4.083, 6.103, 2.839, 2.245, 5.004, 23.95, 6.804, 0.263, 0.062, 1.19, 0.343, 14.249, 0.959, 0.041, 0.134, 4.304, 7.993, 1.922, 0.493, 1.245, 3.995, 1.523, 0.417, 4.349, 1.866, 9.189, 5.617, 9.336, 0.036, 4.94, 0.404, 2.106, 2.105, 0.499, 2.312, 2.333, 1.217, 3.031, 0.189, 7.776, 1.053, 0.155, 14.085, 1.155, 6.527, 4.604, 2.851, 2.388, 0.285, 2.963, 7.984, 0.622, 5.745, 10.474, 2.713, 1.076, 0.357, 0.155, 4.374, 0.211, 1.07, 4.082, 4.45, 9.5, 1.997, 2.646, 7.799, 4.025, 7.721, 6.209, 5.727, 2.295, 8.502, 2.03, 13.98, 0.46, 0.518, 4.831, 25.578, 1.425, 3.08, 3.562, 4.354, 1.359, 0.165, 9.242, 3.81, 4.606, 11.618, 1.513, 0.149, 0.103, 8.577, 3.248, 0.312, 3.104, 3.635, 0.957, 3.27, 4.015, 1.324, 1.657, 11.151, 3.656, 4.845, 1.826, 0.243, 0.645, 1.54, 4.17, 0.507, 7.137, 17.641, 6.212, 0.799, 0.117, 0.589, 3.873, 1.951, 3.625, 7.509, 15.73, 0.849, 12.124, 0.666, 2.699, 0.771, 1.33, 1.789, 1.301, 8.107, 4.051, 37.601, 3.74, 11.417, 0.112, 3.299, 4.708, 2.615, 10.293, 2.296, 1.122, 0.582, 18.197, 0.674, 6.025, 6.15, 0.131, 8.912, 14.352, 6.052, 5.998, 0.412, 0.037, 6.746, 11.599, 0.168, 5.164, 0.794, 0.47, 5.803, 3.607, 4.048, 1.249, 11.631, 1.006, 0.238, 3.776, 0.291, 1.769, 22.424, 2.879, 5.105, 11.034, 7.637, 1, 0.127, 3.558, 25.833, 4.72, 14.95, 2.306, 3.483, 0.636, 2.717, 3.5, 2.282, 0.337, 0.446, 0.543.

## 9 Modified Kies Weibull quantile regression

In some lifetime scenarios, we might require to investigate the impact of some exogenous variables on an endogenous variable. The concept of regression analysis offers researchers an alternative way to undertake such investigations. However, the choice of an appropriate regression model is paramount in order to make reliable inference. When the endogenous variable in question is asymmetric, heavy-tailed or contaminated with outliers, the adoption of a robust regression model such as the quantile regression model (QRM) is vital. In the following, we formulate a new QRM based on the MKWD to study the effect of exogenous variables on a response variable defined on the positive real line.

### 9.1 The quantile regression model

The formulation of the new QRM for modeling a response variable defined on the positive real line is due to its robustness in handling outliers and allowing the estimation of heterogeneous effects of exogenous variables through the evaluation of various quantiles. The MKW QRM is attained by re-parameterization of the distribution in terms of its quantile function (qf) (see [46–48] for more details). Suppose that *Y* is a random variable that follows the MKWD and *η* ∈ (0, ∞) is a quantile parameter. Let *η* = *Q*(*u*) in Eq (9), making *β* the subject from the qf of the MKWD, yields $\beta =\eta^{-\vartheta }\;\text{log}\left(1+{\left(\;\text{log}\left(1/\left(1-p\right)\right)\right)}^{\zeta^{-1}}\right)$, *p* ∈ (0, 1). Inserting *β* into Eq (8) gives us the re-parameterized density in terms of the qf. Thus, the density function of the MKWD is defined in terms of the qf as

$$\begin{array}{c} f\left(y;\vartheta ,\zeta ,\eta \right)=\frac{\zeta \eta^{-\vartheta }\;\text{log}\left(1+{\left(\text{log}\left(1/\left(1-p\right)\right)\right)}^{\zeta^{-1}}\right)\vartheta y^{\vartheta -1}e^{\eta^{-\vartheta }\;\text{log}\left(1+{\left(\text{log}\left(1/\left(1-p\right)\right)\right)}^{\zeta^{-1}}\right)\zeta y^{\vartheta }}}{{\left(1-e^{-\eta^{-\vartheta }\;\text{log}\left(1+{\left(\text{log}\left(1/\left(1-p\right)\right)\right)}^{\zeta^{-1}}\right)y^{\vartheta }}\right)}^{1-\zeta }e^{{\left(e^{\eta^{-\vartheta }\;\text{log}\left(1+{\left(\text{log}\left(1/\left(1-p\right)\right)\right)}^{\zeta^{-1}}\right)y^{\vartheta }}-1\right)}^{\zeta }}},\;y>0. \end{array}$$ (41)

For *p* = 0.10, 0.25, 0.50, 0.75, 0.90 and 0.95, the density function of the 10^*th*^, 25^*th*^, 50^*th*^, 75^*th*^, 90^*th*^ and 95^*th*^ percentiles are attained, respectively.

The MKW QRM is formulated using a monotonically increasing and twice differentiable link function to relate the exogenous variables to the conditional quantiles. Hence, we have

$$b\left(\eta_{i}\right)=z_{i}^{\prime }\tau ,$$

where *b*(⋅) is the link function, *η*_*i*_ is the *i*^*th*^ quantile parameter, ***τ*** = (*τ*_0_, *τ*_1_, …, *τ*_*k*_)′ is the unknown vector of parameters and $z_{i}^{\prime }=\left(1,z_{i1},z_{i2},\ldots ,z_{ik}\right)$ is the unknown *i*^*th*^ vector of exogenous variables. Note that the MKW median regression is obtained when *p* = 0.50. In this paper, the logarithmic link function is used to link the exogenous variables to the conditional quantiles. Hence,

$$\text{log}\left(\eta_{i}\right)=z_{i}^{\prime }\tau ,i=1,2,\ldots ,n.$$

The log-likelihood function for estimating the parameters of the regression model for a sample of size *n* is obtained by substituting *η*_*i*_ into the re-parameterized density function of the MKWD. Hence, the log-likelihood is given by

$$\begin{array}{cc} \ell = & \sum_{i=1}^{n}f\left(y_{i};\vartheta ,\zeta ,\eta_{i}\right). \end{array}$$ (42)

The estimates of the parameters are obtained by directly maximizing the log-likelihood function.

### 9.2 Model diagnostics

Examining the model adequacy is vital when fitting a model to a data set. The residuals obtained from fitting the model to the data set are often checked to ensure that they behave well and thus the model provides an adequate fit to the data. We employ the randomized quantile residuals (RQR) in this study to examine the adequacy of the model. The RQR are given by:

$$e_{i}=\Phi^{-1}\left(F\left(y_{i};\hat{\vartheta },\hat{\zeta },\hat{\tau }\right)\right),i=1,2,\ldots ,n,$$

where Φ^−1^(⋅) is the qf of the standard normal (SN) distribution. If the model offers adequate fit to the data, the RQR are anticipated to follow the SN distribution [49].

### 9.3 Simulation experiments for the QRM

In this subsection, simulation experiments are executed utilizing three different scenarios of parameter combinations in order to evaluate the performance of the ML method in estimating the parameters of the QRM. The parameter combination used for scenarios I, II and III are: I: *τ*_0_ = 0.6, *τ*_1_ = −0.1, *ϑ* = 0.4, *ζ* = 0.5, II: *τ*_0_ = 4.3, *τ*_1_ = 0.3, *ϑ* = 0.2, *ζ* = 1.5 and III: *τ*_0_ = −0.3, *τ*_1_ = −0.1, *ϑ* = 3.2, *ζ* = 0.5. The experiments are accomplished using the conditional median regression. The experiments are repeated 1000 times for each sample size *n* = 25, 100, 250, 500, 800, 1000. During the simulations, we employ the following regression structure:

$$\eta_{i}=\text{exp}\left(\tau_{0}+\tau_{1}z_{i1}\right),i=1,2,\ldots ,n.$$

The exogenous variable, *z*_*i*1_, is generated using the SN distribution and is held fixed in the simulation process. The performance of the ML method is examined using the mean estimate (ME), average absolute bias (AAB), RMSE, coverage probability (CP) of the 95% confidence interval (CI), lower CI (LCI), upper CI (UCI) and average width of the CI (AWCI). From Tables 16–18, it is observed that as the sample size increases, the MEs approaches the true parameter value, the AABs and RMSEs decrease as expected, the 95% CI CPs gets closer to the nominal value of 0.95, the CI becomes narrower and the AWCI decreases. The simulation results suggest that the MLEs are consistent and the ML method is able to estimate the regression parameters well.

**Table 16:** MKW QRM simulation results for scenario I.

Parameters	*n*	ME	AAB	RMSE	CP	LCI	UCI	AWCI
*τ*_0_ = 0.6	25	0.7652	0.7065	0.8561	0.9280	-0.9543	2.4848	3.4391
	100	0.4963	0.3375	0.4392	0.9440	-0.3788	1.3714	1.7502
	250	0.5903	0.2233	0.2826	0.9580	0.0354	1.1453	1.1099
	500	0.5853	0.1581	0.1960	0.9510	0.1943	0.9762	0.7819
	800	0.6072	0.1264	0.1584	0.9380	0.2991	0.9152	0.6161
	1000	0.6016	0.1115	0.1386	0.9600	0.3259	0.8773	0.5514
*τ*_1_ = −0.1	25	-0.0959	0.5251	0.6913	0.8590	-1.2349	1.0431	2.2781
	100	-0.0983	0.2169	0.2733	0.9130	-0.5855	0.3888	0.9743
	250	-0.0988	0.1177	0.1476	0.9450	-0.3931	0.1955	0.5885
	500	-0.0989	0.0872	0.1092	0.9390	-0.3036	0.1058	0.4094
	800	-0.0975	0.0652	0.0793	0.9680	-0.2572	0.0621	0.3193
	1000	-0.1040	0.0608	0.0761	0.9300	-0.2471	0.0390	0.2860
*ϑ* = 0.4	25	0.4901	0.2306	0.3122	0.9650	-0.2048	1.1851	1.3899
	100	0.4243	0.0846	0.1205	0.9690	0.1753	0.6734	0.4981
	250	0.4088	0.0578	0.0720	0.9690	0.2564	0.5613	0.3050
	500	0.4025	0.0414	0.0518	0.9610	0.2979	0.5071	0.2091
	800	0.4052	0.0337	0.0430	0.9530	0.3229	0.4876	0.1646
	1000	0.4030	0.0293	0.0371	0.9560	0.3297	0.4762	0.1465
*ζ* = 0.5	25	0.7918	0.4730	1.0623	0.9090	-2.3811	3.9648	6.3460
	100	0.5205	0.1187	0.3252	0.9490	0.0662	0.9749	0.9087
	250	0.5056	0.0789	0.0975	0.9450	0.2900	0.7213	0.4313
	500	0.5063	0.0576	0.0731	0.9440	0.3588	0.6538	0.2949
	800	0.5004	0.0448	0.0577	0.9430	0.3872	0.6137	0.2265
	1000	0.5016	0.0405	0.0522	0.9430	0.4002	0.6031	0.2029

**Table 17:** MKW QRM simulation results for scenario II.

Parameters	*n*	ME	AAB	RMSE	CP	LCI	UCI	AWCI
*τ*_0_ = 4.3	25	4.3740	0.4904	0.6192	0.9330	3.1955	5.5525	2.3571
	100	4.3452	0.2477	0.2959	0.9800	3.7437	4.9467	1.2030
	250	4.3043	0.1489	0.1884	0.9680	3.9204	4.6882	0.7678
	500	4.3174	0.1097	0.1391	0.9210	4.0480	4.5869	0.5390
	800	4.2836	0.0883	0.1075	0.9600	4.0697	4.4976	0.4280
	1000	4.2979	0.0713	0.0887	0.9730	4.1070	4.4888	0.3818
*τ*_1_ = 0.3	25	0.2922	0.4233	0.5384	0.8140	-0.5631	1.1475	1.7106
	100	0.3002	0.1907	0.2407	0.9160	-0.1323	0.7327	0.8650
	250	0.2988	0.1176	0.1472	0.9370	0.0291	0.5686	0.5395
	500	0.3016	0.0782	0.0977	0.9520	0.1118	0.4913	0.3796
	800	0.2986	0.0609	0.0765	0.9530	0.1471	0.4500	0.3030
	1000	0.2994	0.0555	0.0688	0.9530	0.1644	0.4343	0.2699
*ϑ* = 0.2	25	0.5076	0.3619	0.5366	0.9730	-0.3469	1.3622	1.7091
	100	0.2501	0.1146	0.1589	0.9410	-0.0712	0.5715	0.6426
	250	0.2382	0.0774	0.1030	0.9460	0.0458	0.4306	0.3848
	500	0.2157	0.0516	0.0666	0.9410	0.0815	0.3499	0.2683
	800	0.2087	0.0467	0.0576	0.9280	0.1053	0.3120	0.2067
	1000	0.2049	0.0381	0.0474	0.9470	0.1126	0.2973	0.1847
*ζ* = 1.5	25	1.6138	1.2699	2.0728	0.6450	-7.7622	10.9898	18.7520
	100	1.9579	1.0274	2.4129	0.8660	-5.3917	9.3074	14.6992
	250	1.6108	0.6396	1.2654	0.8410	-0.8106	4.0321	4.8427
	500	1.5594	0.4066	0.6568	0.8760	0.3471	2.7717	2.4246
	800	1.5687	0.3809	0.5338	0.8880	0.6459	2.4914	1.8455
	1000	1.5532	0.3009	0.4065	0.9100	0.7721	2.3344	1.5623

**Table 18:** MKW QRM simulation results for scenario III.

Parameters	*n*	ME	AAB	RMSE	CP	LCI	UCI	AWCI
*τ*_0_ = −0.3	25	-0.2907	0.1049	0.1348	0.8660	-0.5078	-0.0736	0.4342
	100	-0.3078	0.0558	0.0678	0.8990	-0.4188	-0.1968	0.2219
	250	-0.2935	0.0267	0.0335	0.9470	-0.3616	-0.2254	0.1362
	500	-0.2992	0.0206	0.0253	0.9600	-0.3480	-0.2504	0.0976
	800	-0.3013	0.0157	0.0202	0.9290	-0.3399	-0.2627	0.0772
	1000	-0.2983	0.0139	0.0174	0.9490	-0.3326	-0.2639	0.0687
*τ*_1_ = −0.1	25	-0.0971	0.0636	0.0860	0.8770	-0.2439	0.0498	0.2937
	100	-0.1016	0.0262	0.0329	0.9140	-0.1616	-0.0415	0.1201
	250	-0.0998	0.0151	0.0187	0.9540	-0.1363	-0.0632	0.0732
	500	-0.1001	0.0103	0.0130	0.9470	-0.1253	-0.0748	0.0505
	800	-0.1004	0.0083	0.0104	0.9390	-0.1205	-0.0803	0.0402
	1000	-0.1003	0.0074	0.0094	0.9350	-0.1181	-0.0826	0.0355
*ϑ* = 3.2	25	3.9027	1.6938	2.2170	0.9700	-1.6902	9.4957	11.1858
	100	3.4182	0.5944	0.8012	0.9920	1.3617	5.4747	4.1130
	250	3.3189	0.4404	0.5616	0.9850	2.0923	4.5454	2.4531
	500	3.2817	0.3068	0.3939	0.9740	2.4381	4.1253	1.6873
	800	3.2271	0.2504	0.3223	0.9580	2.5687	3.8856	1.3169
	1000	3.2362	0.2138	0.2726	0.9680	2.6492	3.8233	1.1741
*ζ* = 0.5	25	0.7798	0.4457	1.1977	0.9570	-3.0562	4.6158	7.6721
	100	0.4963	0.0978	0.1344	0.9660	0.1476	0.8450	0.6974
	250	0.5065	0.0756	0.1002	0.9350	0.2924	0.7206	0.4282
	500	0.4958	0.0515	0.0654	0.9370	0.3530	0.6385	0.2854
	800	0.5016	0.0433	0.0559	0.9620	0.3877	0.6154	0.2278
	1000	0.5002	0.0362	0.0457	0.9580	0.3996	0.6009	0.2013

### 9.4 Application

The potential of the MKW QRM is exemplified in this subsection. The QRM model is adopted to study the effect of gender on the survival times (in years) up to the inception of hypertension. The details of the data can be found in Anzagra et al. [46]. Anzagra et al. [46] model the data using the Chen Burr-Hatke exponential (CBHE) QRM and identified the 75^*th*^ percentile as the best with *ζ*_1_ = 1022.7740 and *ζ*_2_ = 1033.8910. The data is fitted with the regression structure

$$\eta_{i}=\text{exp}\left(\tau_{0}+\tau_{1}{\text{gender}}_{i}\right),i=1,2,\ldots ,119,$$

where male = 1 and female = 0. Table 19 provides the parameter estimates and information criteria for various quantiles. It shows that “gender” is insignificant. Thus, an individual’s gender has no significant effect on the survival time to the inception of hypertension. The MKW QRM offers a better fit to the data than the CBHE QRM. Among the fitted quantile for the MKW QRM, the 25^*th*^ percentile yielded the best fit with *ζ*_1_ and *ζ*_2_ values given as 1013.1460 and 1024.2620, respectively.

**Table 19:** Parameter estimates for various quantiles and information criteria.

*p*		$\hat{\tau_{0}}$	$\hat{\tau_{1}}$	$\hat{\vartheta }$	$\hat{\zeta }$	*ζ*_1_	*ζ*_2_
0.01	Estimates	2.9769	0.0321	0.0350	87.5309	1016.6010	1027.7170
	Standard error	0.0847	0.0459	0.0024	5.5861 × 10^−6^		
	*p*-value	< 0.0001	0.4773	< 0.0001	< 0.0001		
0.10	Estimates	3.4559	0.0005	0.0392	68.8501	1013.1490	1024.2650
	Standard error	0.0619	0.0496	0.0030	2.3851 × 10^−6^		
	*p*-value	< 0.0001	0.9921	< 0.0001	< 0.0001		
0.25	Estimates	3.7259	0.0005	0.0395	68.5204	1013.1460	1024.2620
	Standard error	0.0453	0.0496	0.0031	2.4681 × 10^−6^		
	*p*-value	< 0.0001	0.9913	< 0.0001	< 0.0001		
0.50	Estimates	3.9611	0.0005	0.0393	68.8010	1013.1480	1024.2650
	Standard error	0.0349	0.0495	0.0031	1.9873 × 10^−6^		
	*p*-value	< 0.0001	0.9914	< 0.0001	< 0.0001		
0.75	Estimates	4.1460	0.0001	0.0159	169.6091	1013.5080	1024.6240
	Standard error	0.0322	0.0497	0.0012	1.6435 × 10^−7^		
	*p*-value	< 0.0001	0.9978	< 0.0001	< 0.0001		
0.9	Estimates	4.2814	0.0004	0.0141	191.7239	1013.5360	1024.6530
	Standard error	0.0341	0.0497	0.0011	1.7281 × 10^−7^		
	*p*-value	< 0.0001	0.9937	< 0.0001	< 0.0001		
0.95	Estimates	4.3513	0.0004	0.0307	87.8966	1013.2780	1024.3950
	Standard error	0.0362	0.0496	0.0024	1.3344 × 10^−6^		
	*p*-value	< 0.0001	0.9936	< 0.0001	< 0.0001		
0.99	Estimates	4.4659	0.0003	0.0226	119.6513	1013.4040	1024.5210
	Standard error	0.0410	0.0496	0.0018	1.0335 × 10^−6^		
	*p*-value	< 0.0001	0.9956	< 0.0001	< 0.0001		

**Fig 12:**
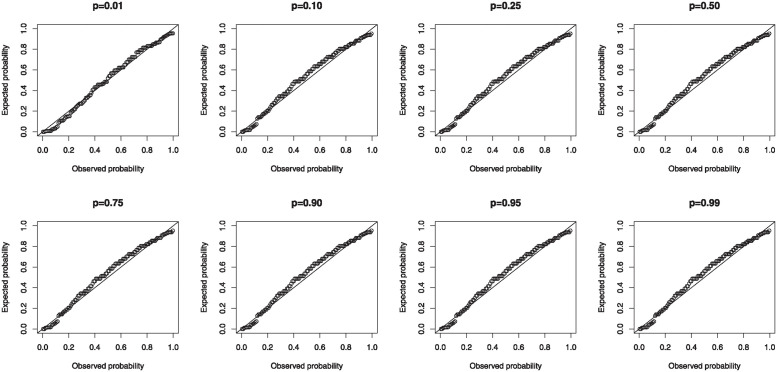
PP plots of RQR for the MKW QRM.

The adequacy of the MKW QRM is evaluated by plotting the PP plots of the RQR, see Fig 12, which confirms that the MKW QRM offers reasonable fit to the data.

## 10 Concluding remarks

This article presented the MKWD, a novel lifetime distribution with three parameters. The statistical properties of the MKWD, including the quantile function, median, moments, mean, variance, skewness, kurtosis, coefficient of variation, moment generating function, incomplete and conditional moments, inequality measures, and order statistics, were computed. Various metrics of entropy and extropy were calculated for the MKWD. The paper examined eight estimation methods to analyze the characteristics of the model parameters for the MKWD. A Monte Carlo simulation was performed to evaluate the effectiveness of these different estimators. The effectiveness of the proposed model was illustrated by analyzing two real data sets. Moreover, the use of survival times data in regression analysis was analyzed by the MKWD, other existing distributions and regression models.

To refer back to the research questions and hypotheses formulated at the beginning:

(1) The MKWD provides a more accurate fit for lifetime data with non-monotonic failure rates compared to various existing Weibull variants.
(2) The MKWD exhibits many statistical characteristics that make it suitable for a wide range of applications in reliability and survival analysis.
(3) MLE demonstrated a better performance than other estimation methods in terms of accuracy and reliability.
(4) The MKWD showed superior modeling performance when applied to real-world datasets, providing better fits and more accurate predictions than competing distributions.

In summary, we have introduced a distribution that is well suited to model lifetime scenarios with non-monotonic failure rates which, due to its superiority over previously considered distributions, is likely to be used in various domains where understanding the lifespan or durability of objects, systems, or processes is crucial. Thus, it can be applied in engineering and reliability analysis, healthcare and medicine, insurance and actuarial sciences, finance and investment, environmental sciences, quality control and manufacturing, telecommunications and networking, and energy and utilities, just to name a few domains. The limitation of our paper is that we only use the complete samples to estimate the parameters of the MKWD. So, for future works, researchers can use the MKWD to estimate its parameters using different censored schemes.

## References

[1] Weibull W. A: statistical distribution function of wide applicability. *J. Appl. Mech*. 1951, 18, 293–297. doi: 10.1115/1.4010337

[2] Luko S. N. (1999). A Review of the Weibull Distribution and Selected Engineering Applications. SAE Transactions, 108, 398–412.

[3] Therneau TM, Grambsch PM. Modeling Survival Data, Extending the Cox Model. Springer: New York, 2000.

[4] Nadarajah S.; Cordeiro G.M.; Ortega E.M.M. The exponentiated Weibull distribution: A survey. *Stat. Pap*. 2013, 54, 839–877. doi: 10.1007/s00362-012-0466-x

[5] Elbatal I.; Aryal G. On the transmuted additive Weibull distribution. *Austrian J. Stat*. 2013, 42, 117–132. doi: 10.17713/ajs.v42i2.160

[6] Al-Babtain A.; Fattah A.A.; Hadi A.N.; Merovci F. The Kumaraswamy-transmuted exponentiated modified Weibull distribution. *Commun. Stat. Simul. Comput*. 2017, 46, 3812–3832.

[7] Alyami S.A.; Elbatal I.; Alotaibi N.; Almetwally E.M.; Okasha H.M.; Elgarhy M. (2022). Topp-Leone Modified Weibull Model: Theory and Applications to Medical and Engineering Data. *Appl. Sci*., 12, 10431. doi: 10.3390/app122010431

[8] Khalil M.G.; Hamedani G.G.; Yousof H.M. The Burr X Exponentiated Weibull Model: Characterizations, Mathematical Properties and Applications to Failure and Survival Times Data. *Pak. J. Stat. Oper. Res*. 2019, 15, 141–160. doi: 10.18187/pjsor.v15i1.2824

[9] Alotaibi N.; Elbatal I.; Almetwally E.M.; Alyami S.A.; Al-Moisheer A.S.; Elgarhy M. Bivariate Step-Stress Accelerated Life Tests for the Kavya-Manoharan Exponentiated Weibull Model under Progressive Censoring with Applications. *Symmetry* 2022, 14, 1791. doi: 10.3390/sym14091791

[10] Afify A.Z.; Kumar D.; Elbatal I. Marshall Olkin Power Generalized Weibull Distribution with Applications in Engineering and Medicine. *J. Stat. Theory Appl*. 2020, 19, 223–237. doi: 10.2991/jsta.d.200507.004

[11] Alotaibi N.; Elbatal I.; Almetwally E.M.; Alyami S.A.; Al-Moisheer A.S.; Elgarhy M. Truncated Cauchy Power Weibull-G Class of Distributions: Bayesian and Non-Bayesian Inference Modelling for COVID-19 and Carbon Fiber Data. *Mathematics* 2022, 10, 1565. doi: 10.3390/math10091565

[12] Elbatal I., Elgarhy M. and Kibria B. M. G. (2021). Alpha Power Transformed Weibull-G Family of Distributions: Theory and Applications. *Journal of Statistical Theory and Applications*, 20(2), 340–354. doi: 10.2991/jsta.d.210222.002

[13] Alahmadi A.A.; Alqawba M.; Almutiry W.; Shawki A.W.; Alrajhi S.; Al-Marzouki S.; Elgarhy M. A New version of Weighted Weibull distribution: Modelling to COVID-19 data. *Discret. Dyn. Nat. Soc*. 2022, 2022, 3994361. doi: 10.1155/2022/3994361

[14] Aldahlan M.A.; Jamal F.; Chesneau C.; Elbatal I.; Elgarhy M. Exponentiated power generalized Weibull power series family of distributions: Properties, estimation and applications. *PLoS ONE* 2020, 15, e0230004. doi: 10.1371/journal.pone.0230004 32196523 PMC7083325

[15] Almarashi A.M.; Jamal F.; Chesneau C.; Elgarhy M. The exponentiated truncated inverse Weibull-generated family of distributions with applications. *Symmetry* 2020, 12, 650. doi: 10.3390/sym12040650

[16] Al-Moisheer A.S.; Elbatal I.; Almutiry W.; Elgarhy M. Odd inverse power generalized Weibull generated family of distributions:Properties and applications. *Math. Probl. Eng*. 2021, 2021, 5082192. doi: 10.1155/2021/5082192

[17] Alkarni S.; Afify A.Z.; Elbatal I.; Elgarhy M. The extended inverse Weibull distribution: Properties and applications. *Complexity* 2020, 2020, 3297693. doi: 10.1155/2020/3297693

[18] Abouelmagd T.H.M.; Al-mualim S.; Elgarhy M.; Afify A.Z.; Ahmad M. Properties of the four-parameter Weibull distribution and its applications. *Pak. J. Stat*. 2017, 33, 449–466.

[19] Hassan A.; Elgarhy M. Exponentiated Weibull Weibull distribution: Statistical Properties and Applications. *Gazi Univ. J. Sci*. 2019, 32, 616–635.

[20] Al-Babtain A.A.; Shakhatreh M.K.; Nassar M.; Afify A.Z. A New Modified Kies Family: Properties, Estimation Under Complete and Type-II Censored Samples, and Engineering Applications. *Mathematics* 2020, 8, 1345. doi: 10.3390/math8081345

[21] Kleiber C. On Lorenz Order with in Parametric Families of Income Distributions. *Sankhya*, *B*, 61 (1999), 514–517.

[22] Zenga M. Inequality curve and inequality index based on the ratios between lower and upper arithmetic means. *Statistica e Applicazioni*, 2007, 4, 3–27.

[23] Rényi, A. (1960). On measures of entropy and information, Proc. 4th Berkeley Symposium on Mathematical Statistics and Probability, 1, 47-561.

[24] Campbell L.L. Exponential entropy as a measure of extent of a distribution. Z. Wahrscheinlichkeitstheorie verw Gebiete 5, 217–225 (1966). doi: 10.1007/BF00533058

[25] Havrda J. and Charvát F. (1967). Quantification method of classification processes, concept of Structural *a*-entropy, *Kybernetika*, 3, 1, 30–35.

[26] Arimoto S. (1971). Information-theoretical considerations on estimation problems, *Information and Control*, 19, 3, 181–194. doi: 10.1016/S0019-9958(71)90065-9

[27] Tsallis C. (1988). Possible generalization of Boltzmann-Gibbs statistics, *Journal of Statistical Physics*, 52, (1-2), 479–487. doi: 10.1007/BF01016429

[28] Lad F., Sanfilippo G. and Agr G. (2015). Extropy: complementary dual of entropy. *Statist. Sci*., 30, 40–58. doi: 10.1214/14-STS430

[29] Balakrishnan N.; Buono F.; Longobardi M. On weighted extropies. *Commun. Stat.-Theory Methods* 2022, 51, 6250–6267. doi: 10.1080/03610926.2020.1860222

[30] Qiu G. and Jia K. (2018). The residual extropy of order statistics. *Stat. Probab. Letters*, 133, 15–22. doi: 10.1016/j.spl.2017.09.014

[31] Fisher R. A. (1922). On the mathematical foundations of theoretical statistics. *Philosophical transactions of the Royal Society of London*. Series A, 222(594-604), 309–368. doi: 10.1098/rsta.1922.0009

[32] Fisher, R. A. (1925, July). Theory of statistical estimation. In Mathematical proceedings of the Cambridge philosophical society (Vol. 22, No. 5, pp. 700-725). Cambridge University Press.

[33] Choi K. and Bulgren W. G. (1968). An estimation procedure for mixtures of distributions. *Journal of the Royal Statistical Society: Series B (Methodological)*, 30(3), 444–460. doi: 10.1111/j.2517-6161.1968.tb00743.x

[34] Kao J. H.K. (1958). Computer methods for estimating Weibull parameters in reliability studies. *IRE Transactions on Reliability and Quality Control*, 1958, 15–22. doi: 10.1109/IRE-PGRQC.1958.5007164

[35] Swain J., Sekhar V., and James R. W. (1988). Least-squares estimation of distribution functions in Johnson’s translation system. *Journal of Statistical Computation and Simulation*, 29(4), 271–297. doi: 10.1080/00949658808811068

[36] J. H. K. Kao, Computer Methods for Estimating Weibull Parameters in Reliability Studies, *IRE Transactions on Reliability and Quality Control*, vol. PGRQC-13, pp. 15-

[37] Kao J. H. K. (1959). A Graphical Estimation of Mixed Weibull Parameters in Life-Testing of Electron Tubes. *Technometrics*, 1(4), 389–407. doi: 10.1080/00401706.1959.10489870

[38] Almalki S. J., Yuan J., A new modified Weibull distribution, *Reliab. Eng. Syst. Safety*, 111 (2013), 164–170. doi: 10.1016/j.ress.2012.10.018

[39] Afify A. Z., Cordeiro G. M., Yousof H. M., Alzaatreh A., Nofal Z. M., The Kumaraswamy transmuted-G family of distributions: Properties and applications, *J. Data Sci*., 14 (2016), 245–270. doi: 10.6339/JDS.201604_14(2).0004

[40] Khan M. S., King R., Hudson I. L., Transmuted modified Weibull distribution: Properties and application, *Eur. J. Pure Appl. Math*., 11 (2018), 362–374. doi: 10.29020/nybg.ejpam.v11i2.3208

[41] Almalki S. J., Yuan J., A new modified Weibull distribution, *Reliab. Eng. Syst. Safety*, 111 (2013), 164–170. doi: 10.1016/j.ress.2012.10.018

[42] Al-Sulami D. (2020). Exponentiated exponential Weibull distribution: mathematical properties and application. *American journal of applied sciences*, 17(1), 188–195. doi: 10.3844/ajassp.2020.188.195

[43] Afify A. Z., Nofal Z. M., Butt N. S., Transmuted complementary Weibull geometric distribution, *Pak. J. Stat. Oper. Res*., 10 (2014), 435–454. doi: 10.18187/pjsor.v10i4.836

[44] Lee C., Famoye F., Olumolade O., Beta-Weibull distribution: Some properties and applications to censored data, *J. Modern Appl. Stat. Methods*, 6 (2007), 173–186. doi: 10.22237/jmasm/1177992960

[45] Hassan A. S., Shawki A. W., & Muhammed H. Z. (2022). Weighted Weibull-G Family of Distributions: Theory & Application in the Analysis of Renewable Energy Sources. *Journal of Positive School Psychology*, 6(3), 9201–9216.

[46] Anzagra L., Abubakari A. G. and Nasiru S. (2023). Chen Burr-Hatke exponential distribution: Properties, regressions and biomedical applications. *Computational Journal of Mathematical and Statistical Sciences*, 2 (1): 80–105. doi: 10.21608/cjmss.2023.190993.1003

[47] Abubakari A. G., Luguterah A. and Nasiru S. (2022). Unit exponentiated Fréchet distribution: actuarial measures, quantile regression and applications. *Journal of the Indian Society for Probability and Statistics*. doi: 10.1007/s41096-022-00129-2

[48] Nasiru S., Abubakari A. G. and Chesneau C. (2022). New lifetime distribution for modeling data on the unit interval: properties, application and quantile regression. *Mathematical and Computational Applications*, 27 (105): 1–27.

[49] Dunn P. K. and Smyth G. K. (1996). Randomized quantile residuals. *Journal of Computational and Graphical Statistics*, 5(3): 236–244. doi: 10.1080/10618600.1996.10474708

